# Host-Adaptive Signatures of H3N2 Influenza Virus in Canine

**DOI:** 10.3389/fvets.2021.740472

**Published:** 2021-10-20

**Authors:** Xueyun Li, Jia Liu, Zengzhao Qiu, Qijun Liao, Yani Peng, Yongkun Chen, Yuelong Shu

**Affiliations:** ^1^School of Public Health (Shenzhen), Sun Yat-sen University, Shenzhen, China; ^2^National Institute for Viral Disease Control and Prevention, Chinese Center for Disease Control and Prevention, Beijing, China

**Keywords:** host-adaptive signatures, IAVs, H3N2, evolution, canine

## Abstract

Wild aquatic birds are the primary natural reservoir of influenza A viruses (IAVs), although a small number of viruses can spill over to mammals and circulate. The focus of IAV infection in mammals was largely limited to humans and swine variants, until the emergence of H3N2 canine influenza viruses (CIVs), which provides new perspective for interspecies transmission of the virus. In this study, we captured 54 canine-adaptive signatures in H3N2 CIVs through entropy computation, which were largely concentrated in the interaction region of polymerase proteins on ribonucleoprotein complex. The receiver operating characteristic curves of these sites showed >95% accuracy in distinguishing between the hosts. Nine of the 54 canine-adaptive signatures were shared in avian–human/equine or equine–canine (PB2-82; PB1-361; PA-277; HA-81, 111, 172, 196, 222, 489), suggesting their involvement in canine adaptation. Furthermore, we found that IAVs can establish persistent transmission in lower mammals with greater ease compared to higher mammals, and 25 common adaptation signatures of H3 IAVs were observed in diverse avian–mammals comparison. There were few human-like residues in H3N2 CIVs, which suggested a low risk of human infection. Our study highlights the necessity of identifying and monitoring the emerging adaptive mutations in companion animals by enhanced surveillance and provides a basis for mammal adaptation of avian influenza viruses.

## Introduction

Based on the surface glycoprotein hemagglutinin (HA) and neuraminidase (NA), influenza A viruses (IAVs) are classified into 18 HA subtypes and 11 NA subtypes (1). Although avian species are the natural reservoir of IAVs, mutations and genetic reassortments can facilitate sporadic infection in mammals. Because of species barriers, most IAVs infections in mammals are “dead-end” infections. On rare occasions, however, IAVs can break the species barrier and establish an independent lineage in mammalian species, as exemplified by seasonal H3N2 influenza virus. The first recorded outbreak of H3N2 influenza was caused by influenza A/Hong Kong/1968 (H3N2) virus in 1968 in Hong Kong. This virus comprised two genes from avian influenza virus (AIV) and six genes from the human influenza virus (H2N2). It was not until the early 21st century that H3N2 made another avian-to-mammal “host jump” in canines, and H3N2 and H3N8 are the two major influenza A subtypes that currently circulate in canine hosts (2, 3).

Both avian-like α-2,3–linked sialic-acid receptors and human-like α-2,6-linked sialic-acid receptors were detected in the endothelial cells of the respiratory tract and other organs of dogs (4), suggesting that they may act as “mixing vessels” for the generation of novel reassorted viruses. Indeed, except for the 2009 pandemic H1N1 and various avian influenza viruses (AIVs) were isolated from canines (5, 6), reassorted viruses between swine origin H1N1 and H3N2 canine influenza viruses (CIVs) were occurred in canines in Guangxi, China (7). The *in vitro* experiments indicated that H3 CIVs preferred to bind α-2,3–linked sialic acids (“avian-like receptors”) yet replicated in primary human nasal and bronchial epithelial cells, which suggested CIVs may pose a risk of infection to humans as well (8).

H3N2 CIVs were first reported in South Korea in 2007 (3) and have since rapidly spread to China and Thailand (9, 10). They originate from avian lineages and undergo mutations that might be responsible for host adaptation (11). However, little is known regarding the amino acid substitutions in H3N2 CIVs that are related to canine adaptation. W222L in HA facilitates H3N2 CIV infection in dogs, and K576E in PB2 enhances replication ability of H3N2 CIVs in mice (12, 13). Another prevalent subtype of influenza in canine is H3N8, which predominantly circulates in America. It derived from avian-origin H3N8 equine influenza virus (EIV), presenting as an “avian–equine–canine” host shift event in influenza virus (14, 15). Despite their diverged evolution, H3N8 CIV and H3N8 EIV appeared phenotypically equivalent (16). Just as in H3N2 CIVs, mutation at 222 position of HA facilitated viral adaption from H3N8 EIV to dogs (17), indicating that HA-222 plays a crucial role in canine adaptation of influenza virus. Thus, a wide range of comparisons between sequences from multiple hosts may provide clues for molecular markers in host tropism of IAVs.

Several techniques were developed to compute the adaptive strategies of IAVs in humans. For instance, information entropy was used to identify characteristic conserved sites in human IAVs (18), and 42 human-adaptive PB2 markers were detected in the seasonal H1N1 and H3N2 viruses (19). A nonhomogeneous phylogenetic model was used to count equilibrium frequencies of amino acids in different hosts and locations, which identified 172 amino acid sites that are strongly related to the avian to human host shift (20). However, a comprehensive adaptive signature mapping of H3N2 influenza virus in mammals, especially canines, was still lacking. In this study, we rebuilt the evolution history of H3N2 CIVs and used entropy to identify mammalian- and canine-adaptive sites in H3N2 influenza viruses.

## Materials and Methods

### Phylogenetic Analysis

The sequences of the individual segments of H3N2 CIVs were downloaded from the NCBI influenza database (https,//www.ncbi.nlm.nih.gov/genomes/FLU/Database/nph-select.cgi) using “full length plus” and “collapse identical sequences” as the filtering parameters (available on January 27, 2021). After Blasting in NCBI, the first 5,000 sequences of IAVs closest to CIVs were downloaded. H3 and N2 sequences were retrieved, and the sequences of internal genes of all subtypes were selected. Redundant sequences were removed using cd-hit-est before aligning with MAFFT v7.222 (21, 22). Low-quality sequences with degenerate base >5 or gap frequency of more than 20 were excluded. The phylogenetic trees were generated with maximum likelihood method and the general time-reversible substitution model using MEGA v7.0.

### Protein Genome Dataset and Alignment

The protein sequences of the eight gene segments of IAVs were downloaded from NCBI influenza database. In the AIV dataset, all available internal protein sequences were downloaded except that for HA and NA, which only comprised H3N2 subtype. The sequences of canine, equine, human, and swine viruses were downloaded using H3N2/H3N8 subtype as the filtering parameter. A total of 1,131,554 protein sequences, including 207,745 from the AIV dataset, 3,992 from CIVs, 6,326 from EIVs, 879,788 from human and 33,703 from swine influenza virus (SIV) dataset, were downloaded. Target sequences <95% of the full-length and AIVs isolated from humans were excluded, and the number of sequences of each protein finally included in the analysis is shown in Table 1. Then, protein sequence alignments were performed with sequence alignment program.

**Table 1:** The number of protein sequences of target finally used in this study.

	**Avian**	**Canine (H3N2)/(H3N8)**	**Equine (H3N8)**	**Human (H3N2)**	**Swine (H3N2)**
PB2	22,742	236/53	175	38,958	2,501
PB1	19,871	222/50	179	40,167	2,039
PB1-F2	12,628	214/43	110	32,452	1,585
PA	12,175	236/53	159	5,930	934
HA	412 (H3N2)/1,421 (H3N8)	235/92	243	66,542	3,921
NP	23,224	252/74	177	40,586	2,589
NA	393 (H3N2)/1,063 (H3N8)	211/89	158	58,732	3,938
M1	23,858	251/189	219	51,988	3,192
M2	23,837	251/203	200	51,833	3,127
NS1	26,378	210/195	242	37,130	2,630
NS2	21,383	199/193	200	21,383	2,439
Total	189,385	2,517/1,234	2,062	445,701	28,895

### Host-Adaptive Signature Prediction

**Figure 1:**
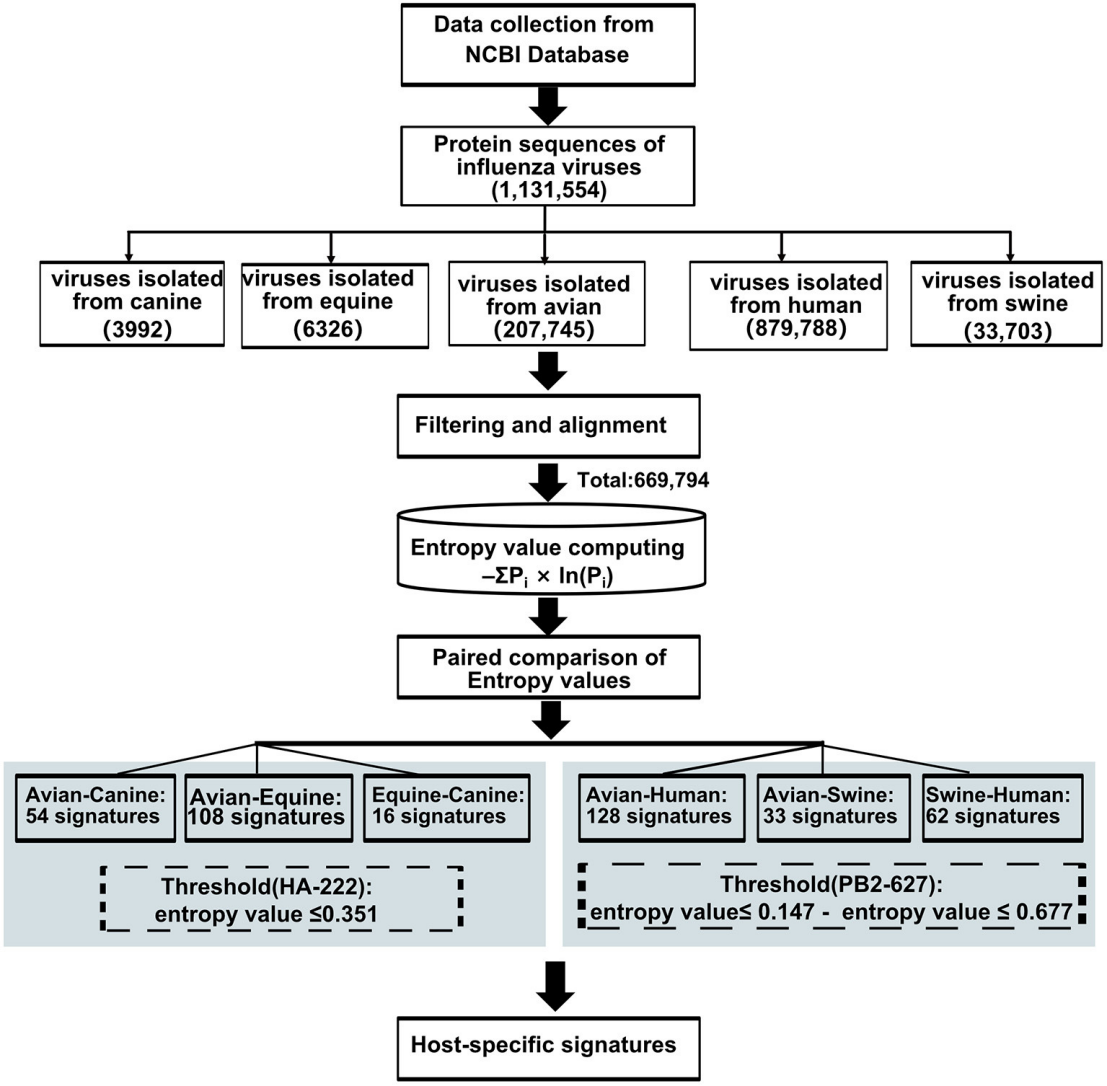
Work flow for predicting host-adaptive signatures.

Forty-seven avian–human signatures were computed using the formula -ΣP_*i*_×ln(P_*i*_), as described by Chen et al. (19) based on Shannon entropy that has been used to evaluate the diversity of a system. The conservation of amino acids was measured by entropy value (X) of dominant amino acid residue in the position, which has experimentally validated cross-species association. An amino acid is defined as a host-adaptive marker if the entropy value of the dominant amino acid at the given site is (i) less than or equal to X (22) and (ii) inconsistent between the two species. The substitution at position 222 in the HA gene of H3N2 AIVs with entropy value of 0.351 was used as the threshold when screening for host-specific signatures between avian and canine/equine viruses. To compare avian and human viruses, the dominant amino acid value at position PB2-627, which is a widely reported species-associated position in various subtypes of IAVs, was selected as the threshold. The entropy value of dominant amino acid in PB2-627 is 0.147 and 0.677 in the AIVs and human influenza viruses, respectively. As applying any one value as the threshold would lead to the loss of meaningful sites or result in too many irrelevant signatures, a position harboring entropy value ≤ 0.147 and ≤ 0.677 in two-host sequence calculation, respectively, were considered the avian–human signature. The same threshold was used for avian–swine/swine–human analysis. The analysis workflow is outlined in Figure 1. All data were calculated using the Python software 3.7.

### Entropy Evaluation, Receiver Operating Characteristic Curves

Receiver operating characteristic (ROC) curves and area under the curve with 95% confidence intervals were used to evaluate the entropy model. The ROC curves were generated by the MedCalc program. The positions of canine-adaptive signatures were used to calculate true-positive pairs and false-positive pairs in AIVs and H3N2 CIV sequences dataset. The accuracy reflected the efficacy of those positions to distinguish between avian-adaptive or canine-adaptive sequences.

## Results

### Phylogenetic Analysis of CIVs

**Figure 2:**
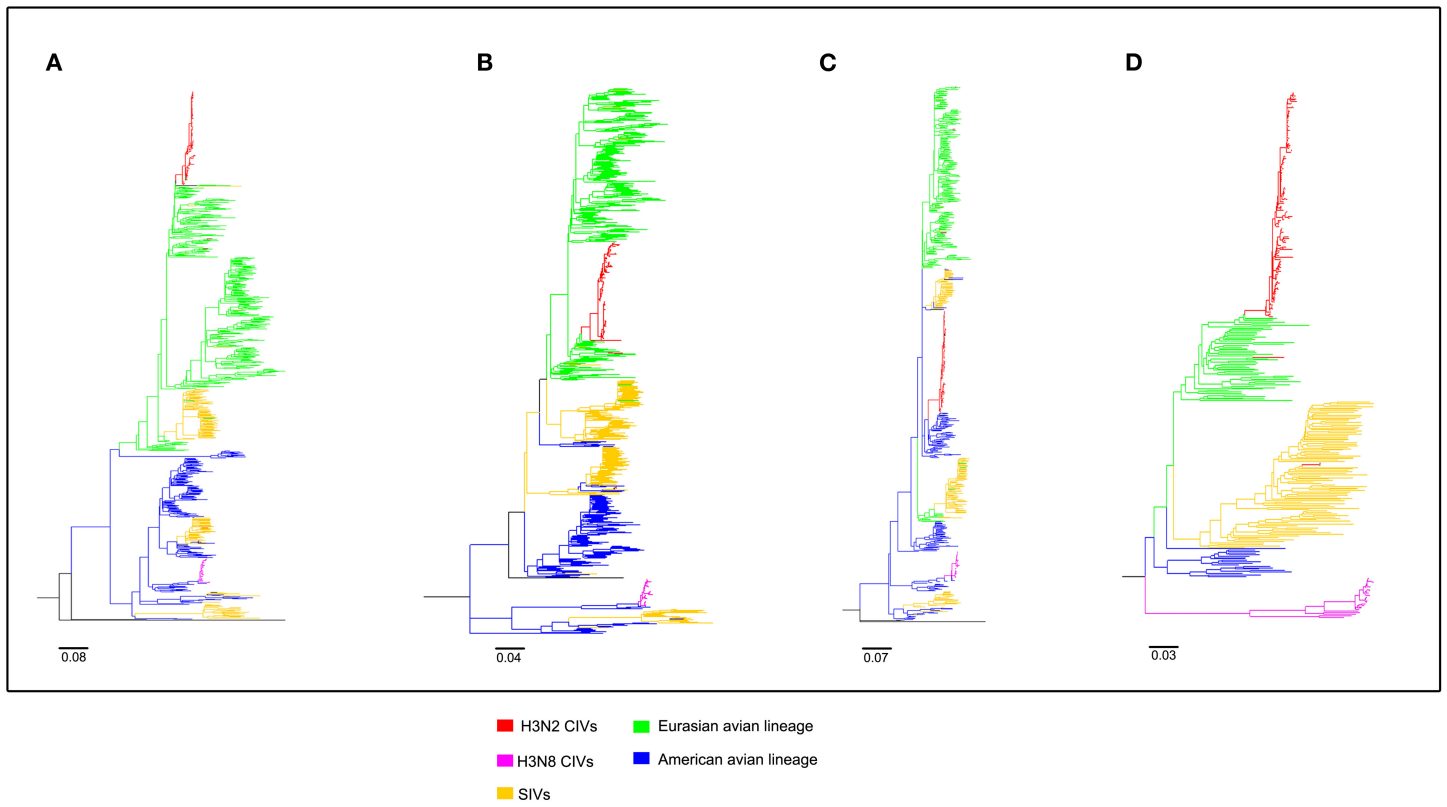
Maximum likelihood trees of segment 1-4 of H3N2 CIVs. **(A)** PB2; **(B)** PB1; **(C)** PA; **(D)** HA. Colored branches represent distinct lineages. H3N2 CIVs: red, H3N8 CIVs: purple, SIVs: yellow, Eurasian avian lineage: green, and American avian lineage: blue. The bars are drawn to scale.

**Figure 3:**
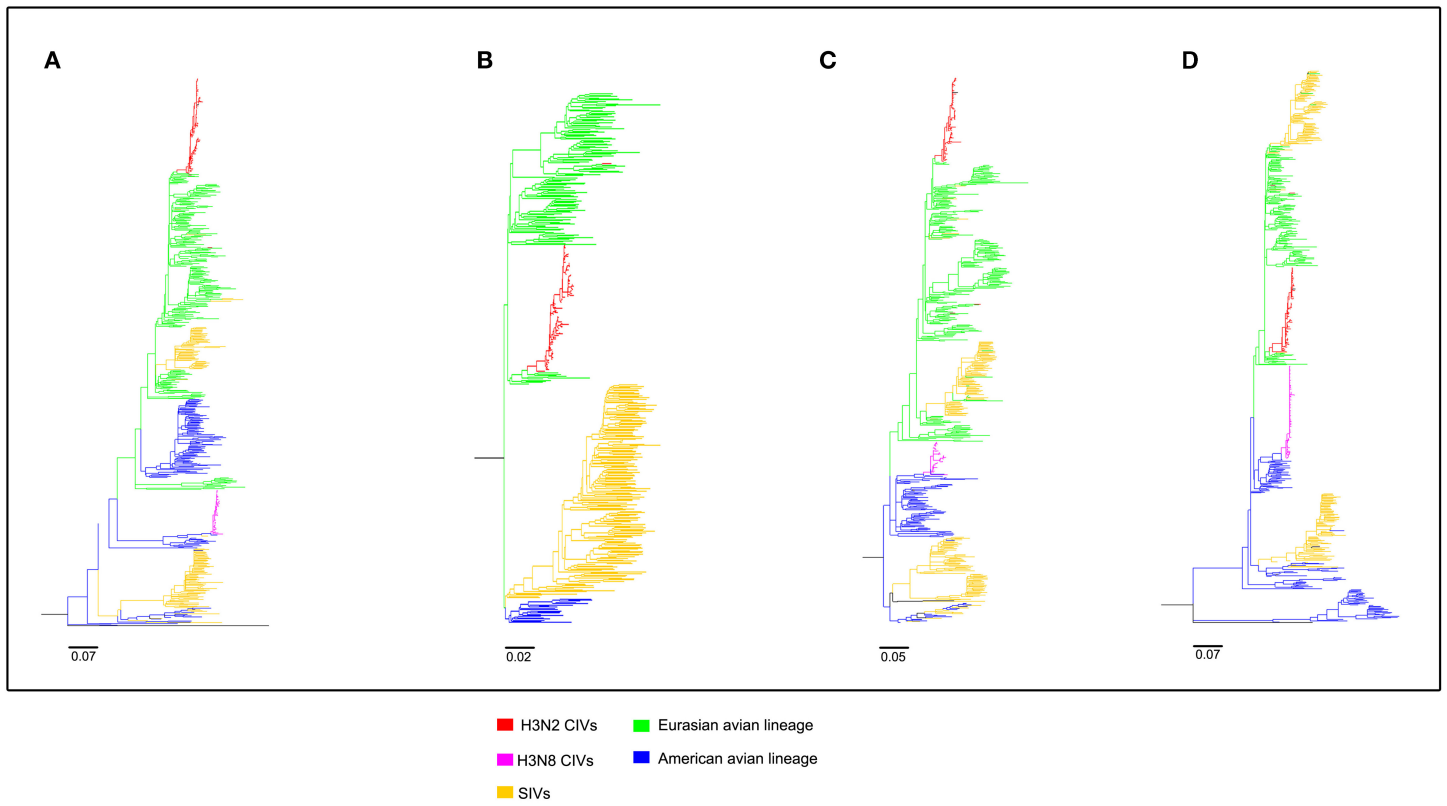
Maximum likelihood trees of segment 5-8 of H3N2 CIVs. **(A)** NP; **(B)** NA; **(C)** M; **(D)** NS. Colored branches represent distinct lineages. H3N2 CIVs: red, H3N8 CIVs : purple, SIVs: yellow, Eurasian avian lineage: green, and American avian lineage: blue. The bars are drawn to scale.

To clearly map the evolutionary background of H3N2 CIVs, the sequences of different avian influenza lineages were included in phylogenetic analyses. As shown in Figures 2, 3, the H3N2 CIVs (red cluster) and H3N8 CIVs (purple cluster) were clustered into two branches respectively, revealing that they have different origins. H3N2 CIVs mainly originated from Eurasian avian viruses and circulated for a long time in Eurasia, HA (Figure 2D) and NA (Figure 3B) segment exhibited a monophyletic origin nested within the H3N2 avian influenza lineage. Consistent with previous studies, we found that the H3N2 CIVs emerged from cross-species transmission of AIVs. In contrast, H3N8 CIVs originated from EIVs.

### Host-Adaptive Signatures of Avian–Canine in H3N2

**Figure 4:**
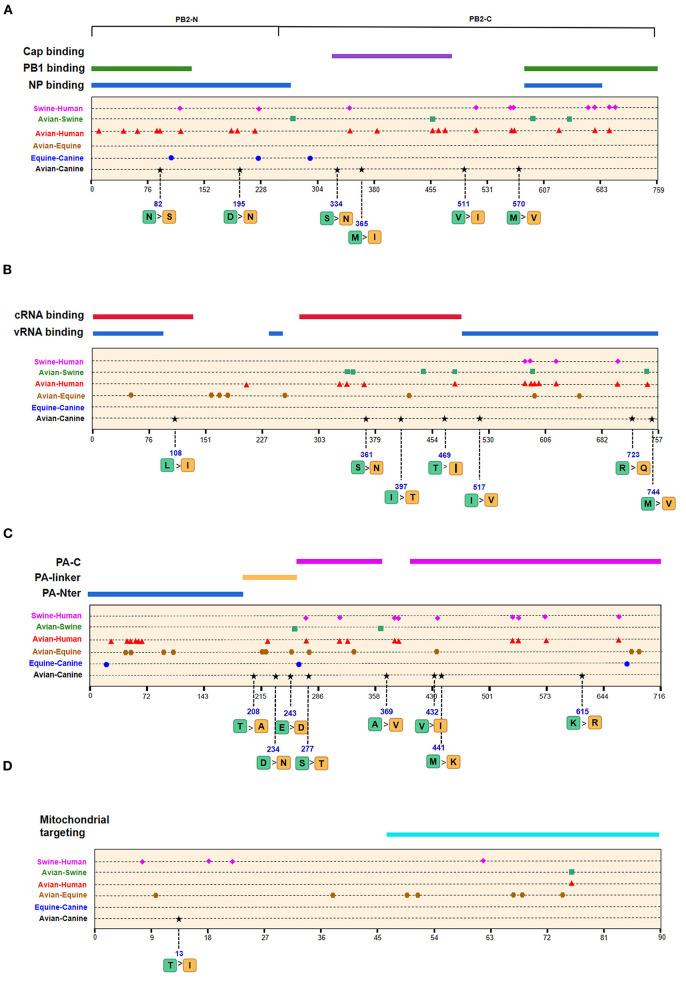
Positions of identified signatures in the structural and functional domains of PB2 **(A)**, PB1 **(B)**, PA **(C)**, and PB1-F2 **(D)** proteins. The positions and dominant amino acid residues of Avian→ Canine (H3N2) signatures are placed below the pale-yellow bar. Black star: Avian→ Canine (H3N2), blue circle: Equine→ Canine (H3N8), brown hexagon: Avian→ Equine (H3N8), red triangle: Avian→ Human (H3N2), green square: Avian→ Swine (H3N2), and purple triangle rhomb: Swine→ Human (H3N2). The detail of these signatures are in Table 2.

We next screened for the species-specific amino acid changes associated with host adaptation in H3N2 CIVs. A total of 54 amino acid signatures separated H3N2 CIVs from AIVs on the basis of the Shannon entropy value of the HA-222 position of H3N2 AIVs (Table 2 and Supplementary Table 1). HA (14/54) was most frequently mutated, followed by NA (9/54) and PA (8/54). H3N2 has consistently circulated in the canine population and spread to other mammals such as humans and swine. We compared the positions of the host-adaptive signatures of H3N2 in canine with these mammals. Five amino acid sites were common to avian–canine (H3N2) and avian–human (H3N2) entropy results, including 82 in PB2, 361 in PB1, and positions 172,196, and 489 in HA protein. These results suggest that adaptive mutations at these sites may be important for AIVs to cross the species barrier and infect mammals. Zhu et al. identified 45 mutations based on the H3N2 CIV lineage and avian lineages (11), of which 31 were identified in our study as well. In addition, 23 novel genomic signatures were also discovered. Previous studies have demonstrated that the most adaptive mutations conferring enhanced polymerase activity are localized in two clusters, the N and C termini of PB2 (especially in NP-binding and the PB1-binding domain of C-terminus), for example, the positions 627 and 701 (23, 24). We identified mutations at sites 82 and 195 located in the N-terminal NP/PB1-binding region of PB2 protein, along with sites 334 and 365 in the C-terminal cap-binding domain. No mutations were detected in the C terminal NP/PB1-binding region (Figure 4A).

**Table 2:** The positions of adaptive amino acid substitutions in different host-pair calculations.

	**Avian–canine (H3N2)**	**Equine–canine (H3N8)**	**Avian–equine (H3N8)**	**Avian–human (H3N2)**	**Avian–swine (H3N2)**	**Swine–human (H3N2)**
PB2	82,195,334,365,511,570	107,221,292	/	9,44,67,81,82,120,194, 199,227,271,353,382, 456,463,475,526,567, 569,627,682,697	271,456,591,645	120,199,353,475,526, 567,569,627,674,682
PB1	108,361,397,469,517,723, 744	/	61,157,164,175,261, 429,587,642	212,327,336,361,486,576, 581,586,587,619,709,741	336,339,433,486, 581,741	576,586,619,709
PB1-F2	13	/	10,38,51,52,68,69,74	76	76	8,18,20,62
PA	208,234,243,277,369,432, 441,615	27,256,675	55,57,99,118,216,217, 244,277,336,437,683, 689	28,55,57,62,65,66,225,268, 311,332,383,385,552, 557,573,668	254,362	311,573
HA	10,45,81,111,128,172, 196,222,232,261,326, 435,489,496	29,54,83,118, **222**,328,483	25,56,63,70,81,83,88, 102,111,121,122,143,146, 160,182,207,244,252, 278,300,304,309,328, 331,347,355,387,442, 464,479,489,541	31,33,48,53,75,172, 186,192,196,198,202, 212,223,225,227, 361,384,452,489,530	/	33
NP	125,159,374,418,428,473	375	41,50,117,146,245,293, 305,312,319,345,351,374, 453,496,498	16,18,31,61,65,100,131, 197,214,280,283,286,293, 305,312,313,343,344,357, 422,423,442,455,459,472	21,119,189,190, 289,305,357,400, 425,444,456	18,52,65,131,214,239, 280,283,286,312, 344,372,406,422,442, 455,459
NA	9,24,54,65,156,208,372, 380,432	62,147	19,22,43,47,82,91,93,160, 199,211,233,250,258, 260,266,301,311,319, 321,339,359,374, 386,393,415,454	18,23,42,152,217,223, 247,249,312,369,374, 389,404,439	/	152,217,247,249,312, 389
M1	/	/	85	115,121,137,174,218,239	116,209,214	115,137,167,174, 218,230,239
M2	/	/	50	54,57,78,86,93	77	57,78,86,89,93
NS1	75,172,230	/	96,156,186,214	41,82,125,135,144,196,	125,189	28,82,135,144,196
NS2	/	/	33,35	57,107	32,34,57	107

*The position of signature shared in CIVs was shown in bold. /, no host-specific amino acid substitutions in the protein*.

**Figure 5:**
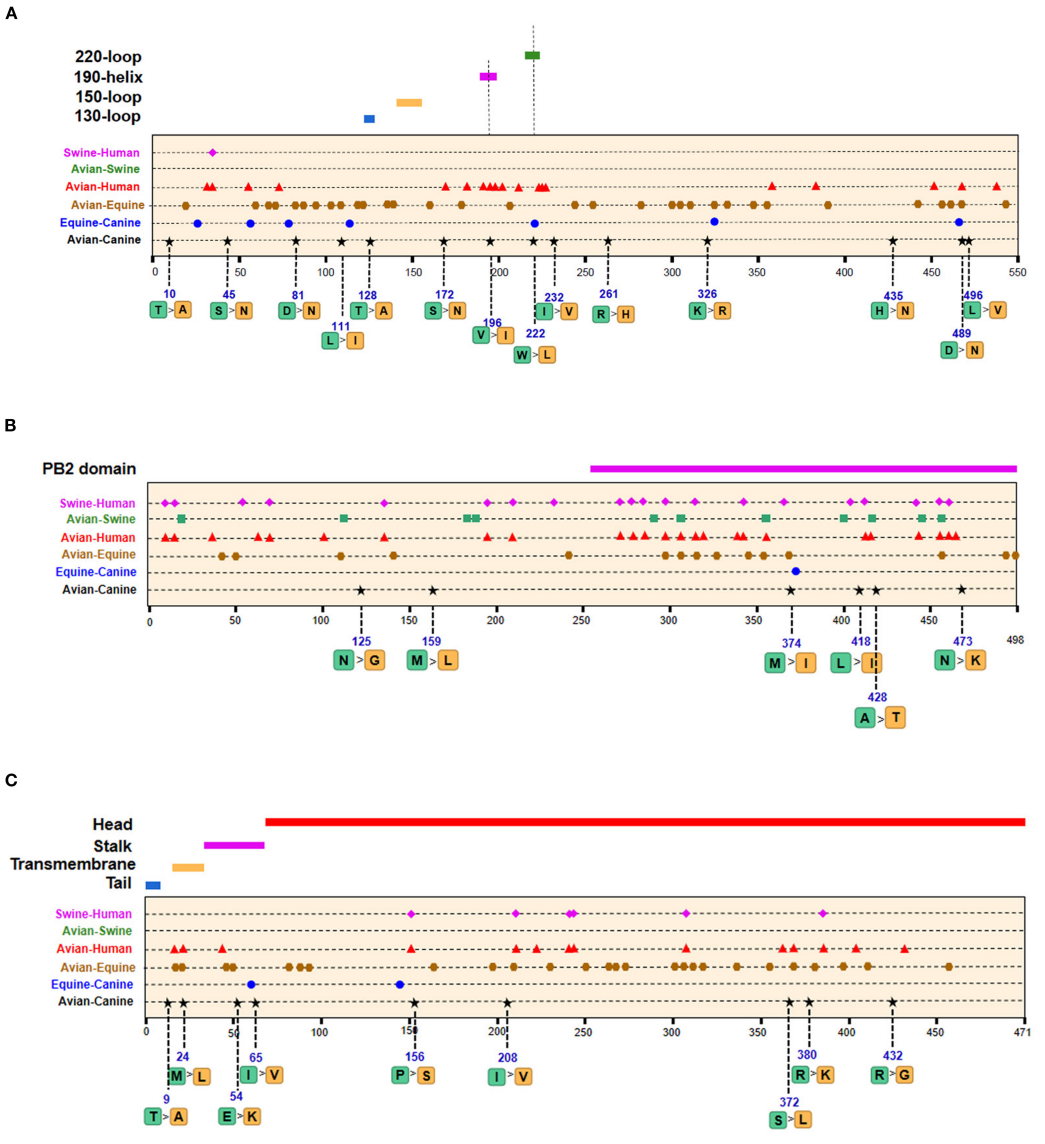
Positions of identified signatures in structural and functional domains of HA **(A)**, NP **(B)**, and NA **(C)** proteins. The positions and dominant amino acid residues of Avian→ Canine (H3N2) signatures are placed below the pale-yellow bar. Black star: Avian→ Canine (H3N2), blue circle: Equine→ Canine (H3N8), brown hexagon: Avian→ Equine (H3N8), red triangle: Avian→ Human (H3N2), green square: Avian→ Swine (H3N2), and purple triangle rhomb: Swine→ Human (H3N2). The detail of these signatures are in Table 2.

Of the seven characteristic sites in PB1, four (108, 361, 397, 469) were located in the cRNA-binding region and three (517, 723, 744) in the vRNA-binding region (Figure 4B). The C-terminal region in the PA subunit has protease activity and plays a critical role in the transcription and replication of influenza ribonucleoprotein (RNP)–encoding genes (25). Several sites were identified in the C-terminal region, including 277, 432, 441 and 615, along with 208, 234, and 243 in the linker domain (Figure 4C). We detected most signatures in HA, with mutations at positions 196 and 222 in the receptor-binding domain (RBD) (Figure 5A). Four of six characteristic sites were located in PB2-binding domain of the NP protein (Figure 5B). NA protein is the second major transmembrane protein responsible for virion release from the surface of infected cells. We identified two signatures (54 and 65) in the stalk region of NA, and the remaining most in the head region (Figure 5C).

### Host-Adaptive Signatures of Equine–Canine in H3N8

**Figure 6:**
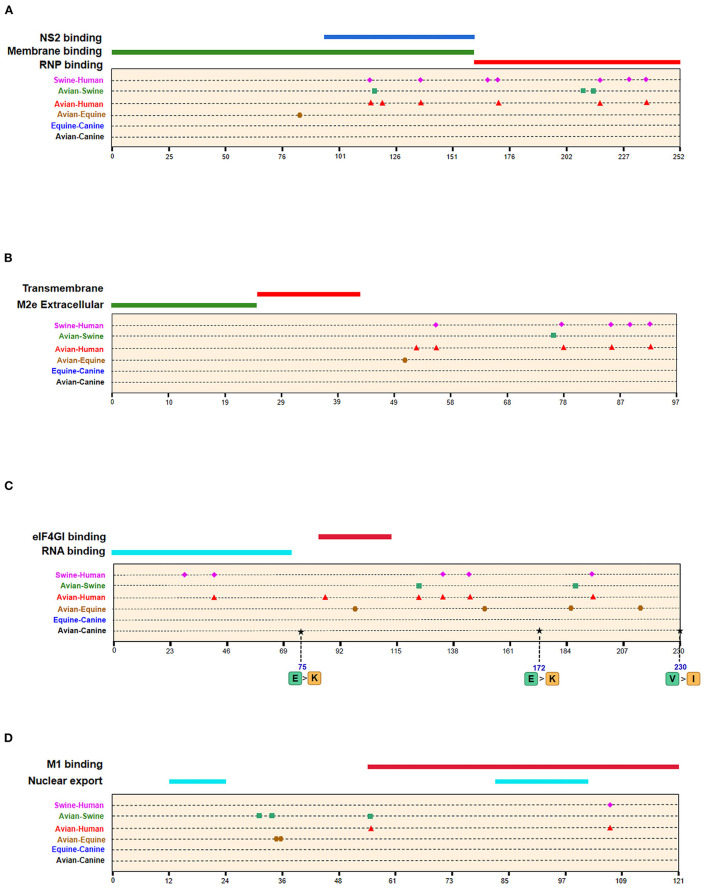
Positions of identified signatures in structural and functional domains of M1 **(A)**, M2 **(B)**, NS1 **(C)**, and NS2 **(D)** proteins. The positions and dominant amino acid residues of Avian→ Canine (H3N2) signatures are placed below the pale-yellow bar. Black star: Avian→ Canine (H3N2), blue circle: Equine→ Canine (H3N8), brown hexagon: Avian→ Equine (H3N8), red triangle: Avian→ Human (H3N2), green square: Avian→ Swine (H3N2), and purple triangle rhomb: Swine→ Human (H3N2). The detail of these signatures are in Table 2.

The emergence of H3N8 CIVs was shown to be a cross-species transmission event of influenza virus from equine to canine (15). To further explore the adaptability of influenza virus in the canine host and the key host-specific signatures in H3N2 CIVs, we further identified 16 signatures that separate H3N8 CIVs from H3N8 EIVs (Table 2 and Supplementary Table 2). The HA contains 7 species-associated amino acid substitutions, of which position 222 was common to the avian–canine and equine–canine comparisons. Three amino acid changes were found in PB2 and PA protein, and one- and two-species–associated signatures were found in NP and NA, respectively. However, no common mutated sites were observed within these four segments compared to H3N2 CIVs. The positions of equine–canine signatures of H3N8 are shown in Figures 4–6 (blue circles). No study, so far, has focused on the equine–canine adaptation sites, although some characteristic sites in AIVs have been verified. For example, the isoleucine (Ile) to valine (Val) mutation at position 292 in H9N2 increased polymerase activity in a mammalian cell line and enhanced virus virulence in mice (26). In addition, the E83K mutation in HA of H5N1 virus facilitates virus binding to α-2,6 receptor (27). The mutation in these sites may also play an important role in equine-to-canine adaptation.

### Host-Adaptive Signatures of Avian–Equine in H3N8

As shown in Table 2 and Supplementary Table 3, the adaptive signatures in avian–equine were much more abundant than in avian–canine (H3N2), indicating greater biological distance between avian and equine. HA contained the most signatures, followed by NA (32/26). Notably, there was not any characteristic site in PB2. Besides, compared to other avian–mammals, the markers in PB1-F2 was the most, suggesting that the selective constraints in PB2 gene may be higher than in other genes; further, PB1-F2 may play a significant role in cross-transmission of AIVs to equine. PA-277 and HA-81,111 were three common signatures between this group and avian–canine. The HA-146 was located in 150-loop of RBD, and its nearby site 143 have an increasing effect on the binding capacity of α-2,6 receptor in H5N1 (27). As reported, the NP-41 could enhance the polymerase activity of AIVs in mammalian cell (28). Such documented sites may also be associated with adaptation of AIVs in equine population.

### Host-Adaptive Signatures of Avian–Human/Swine–Human in H3N2

A total of 128 positions with distinct amino acid residues were identified between human and avian H3N2 influenza viruses (Table 2 and Supplementary Table 4), and most were located in the RNP complex. We obtained fewer signatures compared to a previous study, which can be attributed to different evaluation models used in the studies (29). As shown in Figures 4, 5 most of the characteristic sites in the PB1/NP-binding region of PB2 were similar to that identified in a previous study (30), of which positions 9 and 199 are related to increased virulence of H5N1 in mice (31, 32). In the PB1 protein, most of the adaptation sites were located in the vRNA-binding region. There were 16 human-adaptive signatures in the PA protein, and most were located in the PA-C domain. The mutation at 383 (N→ D) in H5N1 increased the polymerase activity of virus in mammalian and avian cell lines (33). The NP protein harbored the most signatures in all proteins that were mainly concentrated in the PB2 interaction areas. Of the 25 characteristic sites, mutations at positions 357 and 627 in H5N1 are associated with increased virulence in mice (31). The number of human-adaptive sites in other proteins like M1/M2 and NS1/NS2 were fewer, and the positions of these signatures were mainly mapped to the second half part of the target protein (Figure 6).

In addition, we found 62 swine–human host-adaptive signatures (Table 2 and Supplementary Table 6), of which 17 were in NP and 10 were mapped to PB2. The RNP complex contained the most characteristic sites, of which 28 were common to the avian–human and swine–human results. PB2-526, in particular, was associated with increased adaptability of the avian strains to mammalian cell lines (34).

### Host-Adaptive Signatures of Avian–Swine in H3N2

Swine is a “mixing vessel” for the reassortment of influenza viruses, and the ancestor of H3N2 that circulates in swine is of avian origin. In this study, we identified 33 swine-adaptive signatures in H3N2 (Table 2 and Supplementary Table 5), 11 in NP, 6 in PB1, and 4 in PB2. As shown in Figures 4–6, most of these sites were located in the polymerase genome interaction region (green squares). Among the 33 signatures we identified, 4 sites (PB2-271,591;HA-186;NP-357) have been experimentally verified to be associated with host adaptation of influenza viruses to mammals (31, 35–37).

In conclusion, as shown in Table 3, there were 25 common signatures observed in different avian–mammals calculation results of H3 IAVs, most of which were captured through pairwise comparisons, with the largest number in avian–human and avian–swine. In addition, two notable sites were common in triple comparison, with HA-489 in avian–canine/equine/human and NP-305 in avian–equine/human/swine. Among these sites, PB2-271, HA-196, and NP-357 were identified involved in AIV adaption to mammals (31, 35, 38).

**Table 3:** Common signatures in different avian–mammal calculation results of H3 IAVs.

**Avian–mammals comparison**	**Signature**
Avian–canine (H3N2) and avian–human (H3N2)	PB2-82;PB1-361;HA-172,196,489
Avian–canine (H3N2) and avian–equine (H3N8)	PA-277;HA-81,111,489
Avian–equine (H3N8) and avian–human (H3N2)	PB1-587;PA-55,57;HA-489;NP-293,305,312;NA-374
Avian–equine (H3N8) and avian–swine (H3N2)	NP-305
Avian–human (H3N2) and avian–swine (H3N2)	PB2-271,456;PB1-336,486,581,714;PB1-F2-76;NP-305,357;NS1-125;NS2-57
Avian–canine (H3N2), avian–equine (H3N8) and avian–human (H3N2)	HA-489
Avian–equine (H3N8), avian–human (H3N2) and avian–swine (H3N2)	NP-305

### The Risk of H3N2 CIVs to Human

Although no human cases of CIV infection have been reported to date, the risk of zoonotic influenza virus infection needs continuous surveillance and evaluation. To predict the risk of H3N2 CIV infection in humans, we studied the amino acid composition of H3N2 CIVs at positions corresponding to the human-adaptive signatures of H3N2 viruses. As shown in Supplementary Table 4, 123 positions had the same amino acid residues in AIVs and H3N2 CIVs, whereas five positions were distinct. PB2-82 was similar to the human IAV, whereas positions 361 in PB1, 65 in PA, and 172 and 196 in HA exhibited canine-like signatures that were completely distinct from that of avian and human viruses, suggesting a canine-adaptive role of these positions. Given that only few sites in H3N2 CIVs exhibited human-like features at the positions corresponding to avian–human signatures, and based on previous findings (8), we hypothesize that H3N2 CIVs pose a low risk to human. Overall, of the 54 avian–canine signatures identified in H3N2, the amino acid mutations in PB2-82; PB1-361; PA-277; and HA-81,111,172,196,222,489 were common to avian–equine/human or equine–canine signatures and may therefore be important for the canine adaptation of avian viruses.

## Discussion

**Figure 7:**
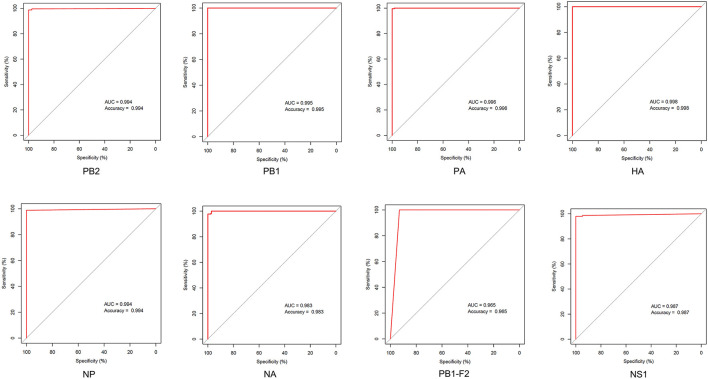
ROC curve of host identification by the avian–canine signatures of different proteins.

Little is known regarding the mammal-specific gene signatures in avian viruses. In this study, we screened for amino acid transitions that are involved in the adaptation of IAVs to canine and other mammalian hosts. The molecular markers of host adaptability are usually identified using phylogenetic and statistical models, which have several disadvantages. The adjusted Rand index can identify distinct sites between the sequences of different hosts, although it is associated with an increase in the false-positive rate (30). Likewise, the results of phylogenetic models are affected if an intermediate host is present, and the selective constraints in this intermediate host are strong (20). In this study, we relied on an entropy threshold to discriminate signatures from nonsignatures as described by Chen et al. Low entropy indicated well-conserved amino acid residues at a site. Although this method has the disadvantage that one single threshold may overlook potential characteristic sites, the substantial sequence data from NCBI database can improve the sensitivity of these calculations. The ROC curves showed that canine-adaptive sites in all proteins except PB1-F2 can distinguish the source sequences with an accuracy higher than 98% (Figure 7). The lower accuracy of PB1-F2 can be attributed to the fewer sites in this protein. Overall, the sensitivity and specificity of identifying host-specific signatures by entropy algorithm were satisfactory.

Currently, H3N2 is the predominant CIV subtype circulating in China. Consistent with previous studies, we identified avian and equine lineages of CIVs. The HA and NA segments of H3N2 CIVs are likely derived from H3N2 avian viruses circulating in Eurasia and the internal segments, originated from Eurasian AIVs. Although we analyzed evolutionary history of H3N2 CIVs, the geographical and seasonal patterns of CIV infection need to be further explored in greater detail.

Adaptive mutation sites were detected in six of the nine internal canine H3N2 proteins, whereas all internal proteins in the human and swine had mutated sites, indicating that the host adaptation of influenza virus is highly complex and requires the entire genomic ensemble. A higher number of signatures indicated greater difficult in transmission and adaption of a viral protein to a new species (30). Our results show that it is more challenging for AIVs to adapt to higher as opposed to lower mammals. For example, there were fewer loci for avian–canine/equine/swine adaptation compared to avian–human adaptation. Notably, some sites were shared in mammal hosts, which raises the possibility that these particular sites carried higher correlation with H3 AIV adaptation to mammals, and HA-489 and NP-305 may deserve more attention. However, it is unclear whether these sites are critical for adaptation or simply coevolve. In addition, the shared sites in different mammalian hosts were few, indicating that the adaptive changes required for AIVs to establish stable lineages vary in different mammals.

We obtained 54 host-specific signatures that distinguish AIVs from H3N2 CIVs by entropy calculating, which encompasses 31 *in silico* markers documented in prior studies, as well as 23 novel markers. However, several proteins of the CIV lack any adaptive mutations, which suggests that the mutations in other proteins are sufficient for the virus to establish a stable linage in canine species. Another possibility is that the number of sequences used to calculate was relatively small, as only limited sequences have been isolated from canine and equine viruses. Besides, in another similar study, 54 characterized genetic substitutions were found to be accumulated and fixed in H3N2 CIV during its circulation in dogs (39). However, there were only five markers observed in our study. Different from the previous study, which calculated the accumulated frequencies of each amino acid over time in CIV sequences, our study explored extremely conservative and inconsistent amino acids sites through the comparison between H3N2 AIVs and CIVs. The divergency of the studies may attribute to the differences in analysis targets and methods.

In this study, we not only elucidated the evolutionary history of H3N2 CIVs but also mapped canine-specific signatures to known functional domain of proteins. As the polymerase of IAV is crucial for replication and transcription, any mutations into these four proteins may improve viral fitness in the new host. We detected six host-restricted sites in PB2 that separated AIVs and H3N2 CIVs, of which two were located in the NP- and PB1-binding domains that regulate RNP assembly and virus replication (40). Several mutations in this region, such as E192K in H5N1 and E158K in H4N6, increase virus replication and virulence in mammals (36, 41). Therefore, the substitutions in 82 and 195 are likely more important compared to other mutations.

We identified seven canine-adaptive signatures in PB1 protein, of which three were located in vRNA-binding region that regulates vRNP complex activity and viral replication. A previous study showed that the SUMOylation-defective K612R mutation in PB1 impaired vRNA binding and activity and inhibited viral replication *in vitro* and *in vivo* (42). It remains to be elucidated whether mutations at positions 517, 723, and 744 affect the pathogenic characteristics of CIVs.

The non-essential viral protein PB1-F2 promotes apoptosis, antagonizes the interferon response, and exacerbates secondary bacterial infections, all of which increase virus virulence (43, 44). We identified a canine-adaptive mutation T13I in this protein. Only a few experimentally verified mammalian adaptive sites are known for this protein, and the N66S substitution in the 1,918 pandemic virus was partly responsible for its high pathogenicity (45). The PA-K615N substitution of H7N7 considerably increases its polymerase activity in mammalian cell lines and increases virulence in mice (46). The K615R located in the C-terminal region of PA protein may play an important role in canine adaption of the virus.

The viral nucleoprotein is crucial for the switch between transcription and replication (47). Some mutations in NP have been identified that are required for the efficient growth of AIVs in mammalian hosts, for example, N319K in H7N7 (46) and K470R in H5N1 (48). Although there were no reported verified sites in our results for AIVs-H3N2 CIVs in NP protein, the virulence marker NP-Q357K known to enhance the pathogenicity of Eurasian H1N1 SIVs (49) was common to avian–human and avian–swine transitions, which is indicative of the reliability of our analysis.

The HA protein initiates influenza virus infection by recognizing and binding to sialylated glycans on the surface of host cells (50). There are four key secondary structure elements in these glycans−150 loop (residues 147–152), 190 helix (residues 190–198), 130 loop (residues 135–138), and 220 loop (residues 221–228) (51). CIVs with mutation W222L in receptor-binding pocket enhanced the binding to canine respiratory receptors (12, 17). Furthermore, H5N1 with Q196R increased virus binding to α-2,6 receptor (52). In this study, V196I and W222L were identified, both of which were located at the receptor binding site and V196I warrants further investigation.

The head domain of the HA molecule is the main target of neutralizing antibodies. T128D is an important determinant of antigenic change during A/H2N2 virus evolution (53). In our study, we found a transition from T to A at position HA-128 after canine adaptation of AIVs, which may result in antigenic changes of the virus.

We obtained nine canine-adaptive signatures in the NA protein, of which five were located in head region. A previous study showed that tolerant substitutions that enabled the NA protein to retain at least 20% of its NA activity are frequently present in the stalk region, indicating that the mutations in the head domain led to NA inactivation. Interestingly, the probability of a mutation leasing to the loss of NA activity decreased with increasing distance from the structural center of the enzyme active site (Y406-N2) (54). Therefore, the mutations at positions 372, 380, and 432 are likely more crucial. The S372A and R403W substitutions in NA enhance the ability of the virus to cross the species barrier and adapt to a mammalian host (55). These substitutions have also been detected in H9N2, H2N2, and H3N2 subtypes (56). Chen et al. demonstrated that mouse-adapted H7N7 virus harbored amino acid changes in the PB2 (E627K), PB1 (R118I), PA (L550M), HA (G214R), and NA (S372N) proteins, which enhanced its ability to replicate in mammalian cells (57). The S372L substitution identified in the NA protein identified in this study may be related to the avian–canine adaptation of AIVs.

Most of the signatures identified in this study were not verified in experimental studies. Furthermore, we were unable to map the mutation sites accurately given the lack of protein structure data currently. We also evaluated the zoonotic transmission risk of H3N2 CIVs, and most signatures exhibited avian–like residues at positions where avian–human signatures were located, which indicate a low risk of H3N2 CIV infection in humans. In summary, we have characterized the adaptive signatures of H3N2 associated with transmission to new mammalian hosts, especially canines. The host-specific sites and canine-adaptive signatures identified by the entropy method exhibited moderate specificity and sensitivity in distinguishing the host source of sequences. It is less challenging for influenza viruses to spread to lower mammals compared to higher mammals, and some common signatures exist in the process of AIV adaption to diverse hosts' environment. The host-adaptation sites on RNP complex are the most abundant and are concentrated in the polymerase proteins interaction domain in canines and other mammals. Of the 54 characteristic sites in H3N2 CIVs, nine were shared between avian–human/equine or equine–canine (PB2-82; PB1-361; PA-277; HA-81,111,172,196,222,489), indicating a crucial role in adapting to canine hosts. Further studies are needed to elucidate the complex mechanisms underlying mammalian adaptation of AIVs.

## Data Availability Statement

The original contributions presented in the study are included in the article/Supplementary Material, further inquiries can be directed to the corresponding authors.

## Author Contributions

XL conceived and wrote the manuscript. JL performed phylogenetic analysis. ZQ performed host-adaptive signatures computing and analysis. QL performed model evaluation and YP collated the data. YC and YS checked and revised the manuscript. All the authors read and approved the final manuscript.

## Funding

This work is supported by the Shenzhen Science and Technology Program (KQTD20180411143323605 to YS), National Mega-projects for Infectious Diseases (2018ZX10305409-004-003 to YC), and Guangdong Province Science and Technology Innovation Strategy Special Fund (2018A030310337 to YC).

## Conflict of Interest

The authors declare that the research was conducted in the absence of any commercial or financial relationships that could be construed as a potential conflict of interest.

## Publisher's Note

All claims expressed in this article are solely those of the authors and do not necessarily represent those of their affiliated organizations, or those of the publisher, the editors and the reviewers. Any product that may be evaluated in this article, or claim that may be made by its manufacturer, is not guaranteed or endorsed by the publisher.
