# Supplementary material for: Host-Adaptive Signatures of H3N2 Influenza Virus in Canine
Source: Front Vet Sci. 2021 Oct 20;8:740472. doi: 10.3389/fvets.2021.740472 (PMC8564371; doi:10.3389/fvets.2021.740472)
Supplement: Supplementary file 3 [file Data_Sheet_3.docx]

Table S1 Amino acid residues separating canine from avian in H3N2

|  | position | Avian | Canine |
| --- | --- | --- | --- |
| PB2 | 82 | D(1)/R(1)/I(1)/V(2)/H(12)/K(29)/T(50)/S(275)/**N**(22382) | N(8)/**S**(228) |
|  | 195 | I(1)/H(5)/S(14)/K(15)/Y(19)/G(146)/E(378)/N(706)/**D**(21452) | D(10)/**N**(226) |
|  | 334 | G(19)/R(17)/N(21)/K(70)/**S**(22609) | S(10)/**N**(226) |
|  | **365** | T(1)/L(15)/V(17)/I(127)/**M**(22577) | M(3)/**I**(233) |
|  | 511 | T(1)/A(2)/M(4)/L(17)/I(1555)/**V**(21163) | V(10)/**I**(226) |
|  | **570** | S(1)/R(2)/K(3)/L(6)/V(65)/I(1654)//**M**(21007) | M(2)/I(1)/**V**(233) |
| PB1 | **108** | R(2)/P(2)/V(10)/F(19)/I(592)/**L**(19246) | **I**(222) |
|  | 361 | V(1)/T(1)/R(26)/G(45)/I(57)/N(78)/**S**(19663) | **N**(222) |
|  | 397 | K(1)/N(1)/R(3)/V(29)/T(88)/L(116)/M(446)/**I**(19187) | **T**(222) |
|  | 469 | S(1)/A(19)/I(25)/**T**(19826) | T(15)/**I**(207) |
|  | **517** | N(5)/T(19)/V(219)/**I**(19628) | I(2)/**V**(220) |
|  | **723** | K(1)/P(1)/G(2)/Q(26)/L(47)/**R**(19794) | R(7)/**Q**(215) |
|  | **744** | K(1)/R(1)/A(4)/V(98)/T(116)/L(171)/I(307)/**M**(19176) | M(12)/**V**(210) |
| PB1-F2 | 13 | S(2)/A(8)/V(35)/P(88)/I(804)/**T**(11691) | **I**(214) |
| PA | **208** | P(1)/N(4)/R(6)/S(8)/I(8)/K(160)/A(178)/**T**(11810) | S(2)/T(9)/**A**(225) |
|  | **234** | N(1)/E(1)/**D**(12173) | D(8)/**N**(228) |
|  | 243 | K(1)/G(2)/D(2)/**E**(12170) | N(2)/E(10)/**D**(224) |
|  | 277 | L(2)/C(4)/T(6)/H(10)/A(16)/Y(36)/P(59)/F(164)/**S**(11878) | H(1)/Y(2)/S(8)/**T**(225) |
|  | **369** | P(1)/S(3)/V(14)/T(79)/**A**(12078) | A(3)/**V**(233) |
|  | **432** | F(1)/A(22)/L(31)/I(1055)/**V**(11066) | V(3)/**I**(233) |
|  | **441** | R(2)/L(3)/I(14)/V(18)/K(20)/T(58)/**M**(12060) | M(3)/R(13)/**K**(220) |
|  | **615** | Q(2)/N(3)/R(211)/**K**(11959) | K(3)/**R**(233) |
| HA | **10** | A(1)/D(5)/M(15)/**T**(391) | V(1)/T(2)/**A**(232) |
|  | 45 | G(1)/N(26)/**S**(385) | S(2)/**N**(233) |
|  | **81** | H(1)/E(2)/G(4)/N(15)/**D**(390) | **N**(235) |
|  | **111** | **L**(412) | L(2)/V(5)/**I**(228) |
|  | 128 | S(1)/N(1)/I(1)/A(34)/**T**(375) | T(15)/**A**(220) |
|  | **172** | N(1)/G(2)/**D**(409) | D(3)/**N**(232) |
|  | 196 | A(2)/I(5)/**V**(405) | A(2)/**I**(233) |
|  | **222** | X(1)/L(43)/**W**(368) | F(1)/W(2)/**L**(232) |
|  | 232 | V(23)/**I**(389) | I(2)/**V**(233) |
|  | 261 | H(5)/Q(13)/**R**(394) | S(1)/R(9)/**H**(225) |
|  | 326 | E(1)/R(20)/**K**(391) | K(7)/**R**(228) |
|  | **435** | **H**(412) | K(1)/H(3)/**N**(231) |
|  | **489** | N(3)/**D**(409) | D(2)/**N**(233) |
|  | 496 | I(5)/V(23)/**L**(384) | L(2)/**V**(233) |
| NP | **125** | H(1)/Y(4)/D(4)/G(4)/T(6)/S(70)/**N**(23135) | S(2)/N(5)/**G**(245) |
|  | **159** | V(1)/L(3)/I(12)/**M**(23208) | M(5)/**L**(247) |
|  | 374 | K(1)/L(12)/V(53)/I(439)/**M**(22719) | M(6)/**I**(246) |
|  | 418 | H(1)/P(1)/F(2)/I(3)/**L**(23217) | L(6)/**I**(246) |
|  | **428** | E(1)/V(2)/S(11)/T(17)/**A**(23193) | A(3)/**T**(249) |
|  | **473** | K(1)/H(3)/T(13)/D(30)/S(88)/**N**(23089) | N(3)/**K**(249) |
| NA | **9** | A(10)**/T**(383) | S(1)/T(3)/**A**(207) |
|  | **24** | **M**(393) | S(1)/M(5)**/L**(205) |
|  | **54** | D(1)/G(1)**/E**(391) | E(5)**/K**(206) |
|  | 65 | M(4)/V(18)/**I**(371) | I(2)/**V**(209) |
|  | **156** | S(1)/**P**(392) | P(5)/**S**(206) |
|  | 208 | V(16)**/I**(377) | I(6)/**V**(205) |
|  | **372** | T(5)/L(14)/**S**(374) | S(5)/**L**(206) |
|  | 380 | G(1)/K(30)**/R**(362) | **K**(211) |
|  | **432** | **R**(393) | R(5)/**G**(206) |
| NS1 | **75** | A(3)/W(5)/V(14)/D(125)/G(126)/K(684)/**E**(26456) | E(5)/**K**(205) |
|  | **172** | G(1)/A(1)/D(1)/Q(10)/K(2907)**E**(24494) | E(5)/**K**(205) |
|  | 230 | L(2)/F(11)/A(17)/I(594)/**V**(21358) | V(2)/**I**(147) |

The positions identified by *Zhu et al*. (2015 ) were shown in bold.

Table S2 Amino acid residues separating canine from equine in H3N8

| Protein | Position | Equine residues | Canine residues |
| --- | --- | --- | --- |
| PB2 | 107 | N(6)/**S**(169) | S(4)/**N**(48) |
|  | 221 | **A**(175) | A(4)/**V**(48) |
|  | 292 | V(1)/**I**(174) | I(4)/**T**(48) |
|  |  |  |  |
|  |  |  |  |
| PA | 27 | E(2)/N(4)/**D**(153) | D(2)/**N**(51) |
|  | 256 | K(4)/**R**(155) | R(4)/**K**(49) |
|  | 675 | **N**(159) | N(1)/**D**(52) |
| HA | 29 | L(5)/**I**(238) | X(1)/I(3)/**M**(88) |
|  | 54 | X(1)/**N**(242) | N(1)/**K**(91) |
|  | 83 | D(1)/T(1)/K(2)/**N**(239) | T(1)/**S**(91) |
|  | 118 | **L**(243) | L(4)/**V**(88) |
|  | 222 | R(1)/L(2)/G(7)/**W**(233) | W(1)/**L**(91) |
|  | 328 | L(7)/**I**(236) | **T**(92) |
|  | 483 | K(1)/**N**(242) | N(1)/**T**(91) |
| NP | 375 | **D**(177) | D(2)/**N**(72) |
| NA | 62 | V(7)/**I**(151) | F(1)/I(6)/**L**(82) |
|  | 147 | I(2)/M(4)/**V**(152) | V(4)/**I**(86) |

Table S3 Amino acid residues separating equine from avian in H3N8

| Protein | Position | Avian residues | Equine residues |
| --- | --- | --- | --- |
| PB1 | 61 | A(1)/S(1)/I(8)/**T**(19861) | T(8)/**I**(171) |
|  | 157 | D(5)/N(17)/I(18)/V(28)/S(135)/T(553)/**A**(19115) | A(1)/**S**(178) |
|  | 164 | L(4)/M(33)/V(35)/**I**(19799) | I(8)/**M**(171) |
|  | 175 | Y(2)/E(9)/G(26)/N(660)/**D**(19174) | D(2)/**N**(177) |
|  | 261 | I(4)/R(8)/N(46)/G(84)/T(104)/A(127)/C(218)/**S**(19280) | S(1)/**R**(178) |
|  | 429 | N(2)/Q(4)/E(8)/R(156)/**K**(19701) | X(1)/K(1)/**R**(177) |
|  | 587 | P(8)/S(26)/V(32)/T(123)/**A**(19682) | A(8)/**T**(171) |
|  | 642 | K(1)/D(1)/I(1)/T(2)/H(2)/Y(4)/S(691)/**N**(19169) | N(3)/**S**(176) |
| PB1-F2 | 10 | R(1)/A(1)/K(1)/I(410)/**T**(12215) | **I**(110) |
|  | 38 | W(1)/S(1086)/**L**(11541) | L(2)/**S**(108) |
|  | 51 | L(1)/I(4)/T(538)/**M**(12085) | M(1)/**T**(109) |
|  | 52 | C(1)/D(2)/L(88)/P(167)/R(241)/**H**(12129) | **P**(110) |
|  | 68 | S(1)/I(463)/**T**(12164) | T(4)/**I**(106) |
|  | 69 | L(55)/P(65)/R(252)/**Q**(12256) | L(1)/**P**(109) |
|  | 74 | N(1)/V(1)/A(29)/P(36)/L(79)/I(410)/**T**(12072) | T(6)/**I**(104) |
| PA | 55 | G(1)/K(4)/N(132)/**D**(12038) | D(7)/**N**(152) |
|  | 57 | G(1)/H(1)/L(1)/W(13)/K(35)/Q(208)/**R**(11916) | M(1)/R(5)/**L**(153) |
|  | 99 | S(1)/D(1)/K(2)/V(6)/E(81)/R(101)/**G**(11983) | G(2)/K(7)/**R**(150) |
|  | 118 | S(1)/M(2)/T(42)/L(101)/V(352)/**I**(11677) | I(9)/**V**(150) |
|  | 216 | B(1)/V(1)/Y(2)/H(3)/S(3)/G(9)/E(61)/N(203)/**D**(11892) | D(9)/**N**(150) |
|  | 217 | G(1)/H(1)/K(1)/P(1)/R(2)/**Q**(12169) | Q(2)/H(6)/**Y**(151) |
|  | 244 | V(1)/S(2)/**G**(12172) | G(8)/**S**(151) |
|  | 277 | L(2)/C(4)/T(6)/H(10)/A(16)/Y(36)/P(59)/F(164)/**S**(11878) | Y(3)/S(8)/**H**(148) |
|  | 336 | P(1)/F(2)/Q(4)/R(7)/M(135)/**L**(12026) | L(3)/**Q**(156) |
|  | 437 | N(1)/Q(7)/Y(110)/**H**(12057) | N(1)/H(7)/**Y**(151) |
|  | 683 | P(1)/F(5)/I(123)/**L**(12046) | L(8)/**I**(151) |
|  | 689 | P(1)/S(10)/**A**(12164) | A(2)/**S**(157) |
| HA | 25 | T(1)/L(10)/M(10)/V(77)/**I**(1323) | F(1)/I(1)/**L**(241) |
|  | 56 | **H**(1421) | H(1)/**Y**(242) |
|  | 63 | X(2)/N(3)/A(5)/E(7)/G(17)/**D**(1387) | D(27)/**N**(216) |
|  | 70 | M(4)/**L**(1417) | L(1)/V(4)/**M**(238) |
|  | 81 | X(1)/E(1)/H(2)/Y(3)/G(6)/N(6)/**D**(1402) | **Y**(243) |
|  | 83 | K(1)/X(1)/A(1)/N(4)/**T**(1414) | D(1)/T(1)/K(2)/**N**(239) |
|  | 88 | L(1)/X(1)/I(26)/**V**(1393) | V(6)**/I**(237) |
|  | 102 | A(1)/M(1)/X(2)/I(7)/**V**(1410) | V(10)/**I**(233) |
|  | 111 | I(1)/X(2)/**L**(1418) | L(7)/**I**(236) |
|  | 121 | V(1)/T(1)/N(1)/X(2)/F(37)/**I**(1379) | F(1)/S(3)/M(12)/**T**(227) |
|  | 122 | I(1)/P(1)/N(1)/X(2)/S(4)/A(97)/**T**(1315) | **A**(243) |
|  | 143 | X(2)/S(4)/**P**(1415) | T(1)/**S**(242) |
|  | 146 | S(1)/X(2)/**G**(1418) | **S**(243) |
|  | 160 | X(2)/S(3)/R(4)/V(5)/T(116)/**A**(1291) | A(1)/**S**(242) |
|  | 182 | X(2)/I(17)/**V**(1402) | T(1)/V(1)/**I**(241) |
|  | 207 | X(2)/K(25)/**R**(1394) | R(1)/E(14)/**K**(228) |
|  | 244 | M(1)/A(1)/X(2)/**V**(1417) | I(2)/V(3)/T(6)/**M**(232) |
|  | 252 | V(1)/X(2)/**I**(1418) | I(19)/**V**(224) |
|  | 278 | X(2)/T(4)/V(45)/**I**(1370) | I(1)/M(1)/W(2)/A(6)/**V**(233) |
|  | 300 | X(2)/V(7)/**I**(1412) | I(9)/**V**(234) |
|  | 304 | S(2)/X(2)/N(4)/V(6)/T(6)/**A**(1401) | A(1)/E(4)/**K**(238) |
|  | 309 | X(2)/I(36)/**V**(1383) | V(14)/**I**(229) |
|  | 328 | X(2)/S(5)/I(5)/P(7)/A(7)/**T**(1395) | L(7)/**I**(236) |
|  | 331 | X(2)/I(19)/**L**(1400) | **I**(243) |
|  | 347 | V(35)/**I**(1386) | I(8)/**V**(235) |
|  | 355 | **H**(1421) | H(6)/**Y**(237) |
|  | 387 | R(56)/**K**(1365) | K(13)/**R**(230) |
|  | 442 | A(4)/**S**(1417) | S(1)/**A**(242) |
|  | 464 | S(2)/**N**(1419) | D(2)/N(14)/**G**(227) |
|  | 479 | X(1)/G(3)/D(39)/**E**(1378) | X(1)/E(17)/**G**(225) |
|  | 489 | G(1)/X(1)/N(9)/E(12)/**D**(1398) | N(1)/D(12)/**Y**(230) |
|  | 541 | K(19)/**R**(1402) | **K**(243) |
| NP | 41 | L(1)/M(1)/A(3)/T(7)/V(617)/**I**(22595) | I(1)/**V**(176) |
|  | 50 | I(1)/R(4)/T(12)/D(20)/G(122)/N(530)/**S**(22535) | G(1)/S(5)/**N**(171) |
|  | 117 | S(2)/T(3)/G(3)/M(4)/K(24)/**R**(23188) | I(1)/R(2)/**M**(174) |
|  | 146 | V(15)/S(36)/T(282)/**A**(22891) | A(1)/**T**(176) |
|  | 245 | N(1)/T(1)/G(68)/**S**(23154) | C(1)/S(4)/**G**(172) |
|  | 293 | I(3)/G(6)/K(174)/**R**(23041) | R(10)/**K**(167) |
|  | 305 | C(1)/S(3)/H(18)/K(264)/**R**(22938) | R(2)/**K**(175) |
|  | 312 | M(1)/A(4)/I(42)/**V**(23177) | V(7)/**I**(170) |
|  | 319 | I(1)/D(1)/M(1)/H(2)/T(18)/S(39)/K(146)/**N**(23016) | N(9)/**K**(168) |
|  | 345 | R(2)/T(3)/N(4)/G(17)/**S**(23198) | S(1)/**N**(176) |
|  | 351 | G(1)/I(3)/K(885)/**R**(22335) | R(1)/**K**(176) |
|  | 374 | K(1)/L(12)/V(53)/I(439)/**M**(22719) | M(2)/**I**(175) |
|  | 453 | E(1)/H(2)/Q(5)/A(7)/L(9)/T(22)/S(130)/**P**(23048) | P(4)/**S**(173) |
|  | 496 | S(1)/T(1)/V(1)/N(1)/C(2)/H(2)/I(3)/L(4)/F(987)/**Y**(22222) | Y(2)/**F**(175) |
|  | 498 | C(1)/T(2)/G(2)/Y(5)/I(6)/H(6)/D(7)/K(9)/S(854)/**N**(22332) | N(8)/**S**(169) |
| NA | 19 | X(1)/T(8)/I(13)/A(19)/**V**(1022) | V(2)/F(4)/**I**(152) |
|  | 22 | T(2)/V(89)/**I**(972) | I(12)/**V**(146) |
|  | 43 | R(5)/K(8)/E(18)/**G**(1032) | R(1)/P(6)/**L**(151) |
|  | 47 | K(3)/G(48)/**E**(1012) | E(1)/R(2)/**G**(155) |
|  | 82 | Y(82)/**F**(981) | **Y**(158) |
|  | 91 | G(1)/N(14)/**D**(1048) | D(1)/**E**(157) |
|  | 93 | N(1)/E(2)/R(44)/**K**(1016) | K(1)/**Q**(157) |
|  | 160 | G(1)/K(4)/**E**(1058) | E(14)/**K**(144) |
|  | 199 | N(4)/R(7)/E(18)/Q(29)/**K**(1005) | K(1)/**Q**(157) |
|  | 211 | A(1)/S(3)/I(4)/**T**(1055) | T(1)/I(1)/**V**(156) |
|  | 233 | K(3)/R(3)/L(6)/**Q**(1051) | Q(1)/**K**(157) |
|  | 250 | K(1)/**Q**(1062) | E(1)/Q(13)/**K**(144) |
|  | 258 | R(2)/L(7)/H(9)/**Q**(1045) | Q(1)/N(10)/**D**(147) |
|  | 260 | R(52)/**K**(1011) | K(13)/**R**(145) |
|  | 266 | V(1)/K(1)/D(91)/**E**(970) | N(1)/**D**(157) |
|  | 301 | I(12)/**V**(1051) | T(2)/V(7)/**I**(149) |
|  | 311 | T(1)/S(8)/Q(13)/K(59)/**R**(982) | R(1)/**T**(157) |
|  | 319 | F(1)/I(25)/**L**(1037) | L(1)/**I**(157) |
|  | 321 | **S**(1063) | S(1)/**T**(157) |
|  | 339 | I(4)/S(13)/V(46)/**M**(1000) | M(1)/**L**(157) |
|  | 359 | V(12)/I(30)/**M**(1021) | M(1)/V(6)/**A**(151) |
|  | 374 | M(3)/I(7)/**L**(1053) | L(7)/**I**(151) |
|  | 386 | D(1)/**E**(1062) | E(1)/**D**(157) |
|  | 393 | I(13)/**V**(1050) | V(1)/**I**(157) |
|  | 415 | G(1)/K(88)/**R**(974) | R(5)/**K**(153) |
|  | 454 | N(1)/**D**(1062) | D(1)/N(5)/**S**(152) |
| M1 | 85 | I(1)/T(9)/D(15)/S(74)/**N**(23759) | N(13)/**S**(206) |
| M2 | 50 | S(1)/R(2)/G(2)/W(3)/F(212)/Y(962)/**C**(22655) | C(11)/**F**(189) |
| NS1 | 96 | S(1)/G(5)/K(10)/D(27)/**E**(27372) | G(3)/E(7)/**D**(232) |
|  | 156 | F(1)/S(1)/T(3)/M(4)/L(7)/V(366)/**I**(27030) | I(6)/**V**(236) |
|  | 186 | V(1)/R(1)/D(3)/G(9)/A(15)/K(85)/**E**(27295) | E(13)/**K**(229) |
|  | 214 | V(1)/R(1)/I(2)/P(10)/H(24)/F(1099)/**L**(26176) | L(2)/**F**(240) |
| NS2 | 33 | P(1)/M(12)/I(113)/**T**(21257) | T(17)/**I**(183) |
|  | 35 | V(2)/S(3)/L(464)/**F**(20914) | F(2)/**L**(198) |

Table S4 Amino acid residues separating human from avian in H3N2

| Protein | Position | Avian residues | Human residues | Dominating residues of H3N2 CIVs in given position |
| --- | --- | --- | --- | --- |
| PB2 | 9 | B(1)/C(1)/S(2)/V(3)/G(24)/E(107)/N(149)/Y(251)/**D**(22211) | A(1)/E(1)/Y(2)/H(3)/G(4)/I(5)/S(11)/T(315)/D(462)/**N**(38154) | D |
|  | 44 | X(1)/D(1)/V(2)/P(3)/T(12)/S(60)**/A**(22685) | T(1)/L(1)/A(380)/**S**(38576) | A |
|  | 67 | F(1)/T(3)/M(3)/L(4)/X(14)/V(310)/**I**(22431) | L(2)/A(4)/I(1259)/**V**(37693) | I |
|  | 81 | L(1)/K(5)/X(12)/S(12)/M(19)/A(101)/I(185)/**T**(22432) | A(1)/S(1)/R(2)/L(8)/V(54)/T(368)/I(751)/**M**(37773) | T |
|  | 82 | R(1)/D(1)/I(1)/V(2)/X(12)/H(15)/K(29)/T(50)/S(275)/**N**(22382) | D(1)/R(5)/I(10)/C(25)/G(26)/T(30)/N(661)/**S**(38200) | S |
|  | 120 | A(1)/K(7)/X(22)/D(62)/G(82)**/E**(22596) | A(2)/N(9)/E(506)/**D**(38441) | E |
|  | 194 | L(8)/X(31)/R(46)/K(109)/H(206)/**Q**(22364) | E(1)/H(3)/L(3)/Q(695)/**R**(38256) | Q |
|  | 199 | R(4)/V(4)/D(6)/X(28)/S(53)/T(80)/**A**(22589) | Y(1)/F(1)/T(2)/A(370)/**S**(38584) | A |
|  | 227 | L(1)/M(25)/A(26)/X(32)/I(118)/**V**(22561) | L(1)/T(6)/M(46)/V(625)/**I**(38280) | V |
|  | 271 | S(1)/V(40)/X(41)/A(76)/M(83)/I(263)/**T**(22258) | V(2)/T(6)/S(19)/**A**(38931) | T |
|  | 353 | Q(1)/N(1)/E(4)/X(29)/R(296)/**K**(22435) | G(1)/N(1)/I(3)/K(4011)/**R**(34942) | K |
|  | 382 | L(1)/F(1)/T(2)/X(32)/V(493)/**I**(22237) | A(1)/I(472)/**V**(38485) | I |
|  | 456 | R(1)/I(2)/Y(6)/K(6)/X(19)/H(28)/S(195)/D(248)/**N**(22258) | C(1)/D(1)/G(2)/R(4)/I(7)/N(559)/**S**(38384) | N |
|  | 463 | T(2)/X(20)/L(39)/M(88)/V(299)/**I**(22315) | L(9)/M(12)/I(666)/**V**(38271) | I |
|  | 475 | S(1)/W(5)/I(10)/X(20)/M(102)/**L**(22624) | I(9)/L(467)/**M**(38482) | L |
|  | 526 | T(4)/N(5)/X(11)/R(471)/**K**(22272) | K(552)/**R**(38406) | K |
|  | 567 | C(1)/A(1)/H(1)/Y(3)/V(4)/X(5)/G(19)/N(64)/E(86)/**D**(22580) | T(1)/K(9)/S(24)/D(399)/**N**(38525) | D |
|  | 569 | P(1)/N(1)/K(1)/I(3)/M(4)/X(11)/S(11)/A(55)/**T**(22677) | E(2)/S(93)/T(714)/**A**(38149) | T |
|  | 627 | A(1)/Q(3)/G(5)/X(9)/V(129)/K(453)/**E**(22162) | T(1)/R(14)/E(376)/**K**(38567) | E |
|  | 682 | A(1)/X(2)/C(2)/D(4)/E(4)/R(5)/S(49)/**G**(22694) | R(1)/I(1)/C(4)/N(96)/G(497)/**S**(38359) | G |
|  | 697 | M(1)/P(2)/F(5)/I(26)/**L**(22725) | V(3)/M(12)/L(1035)/**I**(37908) | L |
| PB1 | 212 | A(9)/I(20)/M(21)/V(23)/**L**(19798) | L(38)/X(38)/M(843)/**V**(39246) | L |
|  | 327 | G(1)/W(1)/K(140)/**R**(19729) | X(32)/R(269)/**K**(39864) | R |
|  | 336 | A(65)/I(110)/**V**(19696) | T(1)/G(2)/X(32)/V(163)/**I**(39967) | V |
|  | 361 | V(1)/T(1)/R(26)/G(45)/I(57)/N(78)/**S**(19663) | G(4)/I(5)/X(35)/S(124)/K(971)/**R**(39026) | N |
|  | 486 | G(1)/P(1)/Q(1)/L(2)/K(107)/**R**(19759) | N(1)/X(11)/R(269)/**K**(39885) | R |
|  | 576 | M(31)/I(58)/**L**(19782) | V(4)/T(5)/X(8)/M(9)/L(3539)/**I**(36601) | L |
|  | 581 | N(1)/K(22)/G(24)/D(321)/**E**(19503) | V(2)/X(6)/G(16)/N(38)/E(289)/**D**(39816) | E |
|  | 586 | M(1)/N(1)/T(1)/Q(1)/R(168)/**K**(19699) | X(10)/K(3039)/**R**(37118) | K |
|  | 587 | P(8)/S(26)/V(32)/T(123)/**A**(19682) | X(3)/S(4)/P(4)/V(16)/I(32)/A(4905)/**T**(35203) | A |
|  | 619 | T(1)/S(1)/H(1)/A(1)/V(2)/Y(2)/G(39)/N(68)/E(201)/**D**(19555) | A(1)/E(5)/X(6)/T(8)/K(52)/S(116)/D(2089)/**N**(37888) | D |
|  | 709 | I(59)/**V**(19812) | X(11)/V(1876)/**I**(38279) | V |
|  | 741 | G(2)/N(2)/D(3)/V(19)/S(77)/T(258)/**A**(19510) | V(1)/X(4)/F(4)/Y(10)/T(17)/A(141)/**S**(39989) | A |
| PB1-F2 | 76 | A(110)/**V**(12518) | X(1)/V(196)/**A**(32255) | V |
| PA | 28 | H(1)/R(2)/Q(2)/T(6)/S(11)/L(14)/**P**(12139) | Q(1)/V(1)/X(3)/M(3)/P(14)/S(129)/**L**(5779) | P |
|  | 55 | G(1)/K(4)/N(132)/**D**(12038) | X(1)/D(138)/**N**(5791) | D |
|  | 57 | G(1)/L(1)/H(1)/W(13)/K(35)/Q(208)/**R**(11916) | K(2)/L(6)/R(13)/**Q**(5909) | R |
|  | 62 | Y(1)/M(3)/L(5)/T(9)/V(294)/**I**(11863) | L(1)/X(2)/I(253)/M(261)/**V**(5413) | I |
|  | 65 | L(5)/T(11)/V(14)/A(16)/Y(16)/P(38)/F(87)/**S**(11988) | F(1)/P(4)/I(5)/S(139)/**L**(5781) | H |
|  | 66 | C(1)/R(1)/N(1)/V(1)/E(7)/D(34)/S(128)/**G**(12002) | N(3)/G(147)/**D**(5780) | G |
|  | 225 | R(2)/G(6)/C(15)/N(24)/**S**(12128) | G(2)/S(148)/**C**(5780) | S |
|  | 268 | P(1)/V(2)/F(27)/I(29)/**L**(12116) | X(1)/V(2)/L(145)/**I**(5782) | L |
|  | 311 | T(1)/V(4)/L(26)/I(46)/**M**(12098) | X(1)/V(1)/T(2)/L(10)/M(634)/**I**(5282) | M |
|  | 332 | H(4)/T(15)/L(18)/S(193)/**P**(11945) | X(1)/I(1)/L(3)/A(5)/P(307)/T(623)/**S**(4990) | P |
|  | 383 | V(3)/Y(5)/G(6)/E(11)/N(13)/**D**(12137) | T(2)/S(10)/D(148)/**N**(5770) | D |
|  | 385 | I(1)/E(1)/N(2)/Q(8)/R(235)/**K**(11928) | S(1)/K(151)/**R**(5778) | K |
|  | 552 | G(1)/I(1)/S(2)/N(3)/A(82)/**T**(12086) | I(1)/R(1)/G(2)/N(4)/T(139)/**S**(5783) | T |
|  | 557 | E(1)/L(3)/A(4)/I(25)/M(29)/**V**(12113) | T(1)/V(156)/M(631)/**I**(5142) | V |
|  | 573 | T(8)/V(43)/**I**(12124) | X(2)/I(954)/**V**(4974) | I |
|  | 668 | L(1)/V(29)/**I**(12145) | L(1)/I(922)/**V**(5007) | I |
| HA | 31 | N(1)/G(2)/**D**(409) | Y(2)/K(2)/V(3)/H(9)/T(18)/D(465)/S(1327)/**N**(64716) | D |
|  | 33 | L(1)/P(1)/**Q**(410) | Y(1)/I(2)/K(5)/D(7)/L(10)/N(15)/H(124)/Q(10419)/**R**(55959) | Q |
|  | 48 | M(1)/K(1)/A(2)/I(3)/**T**(405) | P(2)/G(2)/S(8)/L(15)/V(29)/K(29)/R(85)/A(210)/M(271)/T(8995)/**I**(56896) | T |
|  | 53 | S(2)/**N**(410) | B(1)/V(1)/K(1)/H(2)/A(2)/L(3)/C(8)/S(8)/Y(27)/G(78)/E(250)/N(4835)/**D**(61326) | N |
|  | 75 | L(1)/**H**(411) | C(2)/S(3)/N(4)/K(4)/P(8)/R(9)/L(11)/H(2216)/**Q**(64285) | H |
|  | 172 | N(1)/G(2)/**D**(409) | A(1)/Q(1)/N(8)/K(19)/G(439)/D(799)/**E**(65275) | N |
|  | 186 | G(13)/**S**(399) | E(1)/T(4)/N(6)/P(8)/I(14)/R(19)/A(65)/V(228)/D(763)/S(1539)/**G**(63895) | S |
|  | 192 | **T**(412) | K(1)/A(2)/S(7)/Q(8)/L(10)/F(46)/N(64)/V(202)/T(1966)/**I**(64236) | T |
|  | 196 | A(2)/I(5)/**V**(405) | D(1)/Q(2)/Y(8)/T(14)/S(15)/I(32)/V(950)/**A**(65520) | I |
|  | 198 | S(1)/V(1)/T(2)/**A**(408) | K(1)/E(4)/L(10)/Q(22)/T(36)/P(3292)/A(8172)/**S**(55005) | A |
|  | 202 | I(2)/**V**(410) | R(2)/K(6)/T(21)/V(1880)/**I**(64633) | V |
|  | 212 | **T**(412) | I(1)/L(1)/N(2)/K(3)/V(8)/Q(8)/S(243)/T(7881)/**A**(58395) | T |
|  | 223 | I(2)/**V**(410) | E(1)/A(1)/G(2)/L(2)/T(3)/D(3)/M(6)/W(6)/R(9)/V(7736)/**I(**58773) | V |
|  | 225 | **G**(412) | T(1)/H(1)/A(1)/S(4)/I(4)/E(5)/B(8)/R(8)/G(1841)/N(12241)/**D**(52428) | G |
|  | 227 | P(1)/**S**(411) | I(2)/R(3)/H(5)/L(7)/S(2339)/**P**(64186) | S |
|  | 361 | **T**(412) | S(3)/V(5)/K(19)/M(21)/G(22)/I(1489)/T(2508)/**R**(62475) | T |
|  | 384 | I(2)/**V**(410) | M(1)/W(1)/F(2)/R(8)/I(20)/V(462)/**L**(66048) | V |
|  | 452 | K(8)/**R**(404) | S(1)/Q(2)/N(5)/E(9)/T(9)/R(1366)/**K**(65150) | R |
|  | 489 | N(3)/**D**(409) | B(1)/G(6)/A(6)/V(10)/E(15)/K(15)/T(30)/H(42)/S(47)/Y(71)/D(16001)/**N**(50298) | D |
|  | 530 | M(1)/F(1)/A(2)/**V**(408) | D(1)/F(2)/I(3)/G(3)/S(8)/L(10)/T(41)/V(7853)/**A**(58621) | V |
| NP | 16 | C(1)/A(1)/V(2)/D(15)/N(23)/S(509)/**G**(22673) | V(1)/S(5)/G(230)/**D**(40350) | G |
|  | 18 | R(1)/V(1)/A(1)/K(5)/G(8)/D(106)/**E**(23102) | G(3)/E(946)/**D**(39637) | E |
|  | 31 | G(6)/K(42)/**R**(23176) | R(442)/**K**(40144) | R |
|  | 61 | R(1)/T(8)/L(12)/V(21)/M(199)/**I**(22983) | M(9)/I(234)/**L**(40343) | I |
|  | 65 | S(1)/G(1)/K(15)/**R**(23207) | R(799)/**K**(39787) | R |
|  | 100 | I(15)/V(37)/K(74)/**R**(23098) | R(1)/T(2)/L(3)/F(3)/M(3)/A(51)/I(1977)/**V**(38546) | R |
|  | 131 | L(1)/G(1)/P(3)/V(4)/T(10)/S(32)/**A**(23173) | T(3)/Y(6)/P(18)/R(23)/A(2776)/**S**(37760) | A |
|  | 197 | M(1)/T(3)/V(120)/**I**(23100) | I(5543)/**V**(17681) | I |
|  | 214 | N(1)/L(1)/K(464)/**R**(22758) | R(253)/**K**(40333) | R |
|  | 280 | G(1)/A(6)/M(9)/I(33)/**V**(23175) | L(1)/T(5)/V(2492)/**A**(38088) | V |
|  | 283 | F(1)/P(11)/I(40)/**L**(23172) | Y(1)/H(1)/S(2)/L(237)/**P**(40345) | L |
|  | 286 | T(1)/P(3)/L(4)/S(5)/G(24)/V(114)/**A**(23073) | A(489)/**S**(40097) | A |
|  | 293 | I(3)/G(6)/K(174)/**R**(23041) | R(260)/**K**(40326) | R |
|  | 305 | C(1)/S(3)/H(18)/K(264)/**R**(22938) | R(32)/**K**(40554) | R |
|  | 312 | M(1)/A(4)/I(42)/**V**(23177) | L(1)/M(13)/V(2279)/**I**(38293) | V |
|  | 313 | I(8)/Y(9)/V(13)/S(14)/L(108)/**F**(23072) | C(1)/H(5)/V(22)/F(213)/**Y**(40345) | F |
|  | 343 | A(1)/L(3)/M(3)/I(85)/**V**(23132) | I(2)/V(404)/**L**(40180) | V |
|  | 344 | A(1)/F(1)/P(1)/T(5)/L(183)/**S**(23033) | F(1)/S(241)/**L**(40344) | S |
|  | 357 | T(1)/E(1)/L(3)/H(4)/R(6)/K(73)/**Q**(23136) | T(1)/Q(2)/R(104)/**K**(40479) | Q |
|  | 422 | T(1)/G(2)/K(7)/**R**(23214) | Q(1)/M(1)/R(240)/**K**(40344) | R |
|  | 423 | G(1)/E(1)/V(70)/S(118)/T(323)/**A**(22711) | Q(1)/L(2)/T(19)/P(144)/A(238)/**S**(40182) | A |
|  | 442 | I(3)/A(16)/S(31)/**T**(23174) | T(243)/**A**(40343) | T |
|  | 455 | R(1)/V(1)/A(1)/G(2)/Y(6)/N(17)/E(35)/**D**(23161) | D(245)/**E**(40341) | D |
|  | 459 | L(1)/K(1)/R(4)/H(26)/**Q**(23192) | W(2)/K(7)/Q(376)/**R**(40201) | Q |
|  | 472 | K(1)/R(1)/M(1)/S(2)/A(123)/**T**(23096) | S(2)/T(14823)/**A**(25761) | T |
| NA | 18 | T(1)/**A**(392) | V(1)/L(1)/P(1)/I(1)/W(2)/C(2)/F(2)/G(2)/Y(3)/T(32)/A(2272)/**S**(56413) | A |
|  | 23 | F(2)/**L**(391) | Y(1)/Q(1)/M(1)/I(2)/V(5)/S(35)/L(2493)/**F**(56194) | L |
|  | 42 | X(1)/F(1)/Y(3)/**C**(388) | R(1)/W(2)/P(3)/N(3)/I(4)/V(8)/Y(23)/S(38)/L(91)/C(2313)/**F**(56246) | C |
|  | 152 | K(1)/**H**(392) | N(1)/K(2)/G(3)/C(14)/D(14)/L(79)/S(179)/H(4233)/**R**(54207) | H |
|  | 217 | M(1)/V(3)/**I**(389) | H(1)/G(3)/S(3)/L(4)/T(4)/A(5)/I(4326)/**V**(54386) | I |
|  | 223 | **N**(393) | Y(1)/R(1)/S(2)/L(3)/G(12)/I(16)/N(1068)/K(1808)/E(8731)/**D**(47090) | N |
|  | 247 | N(6)/**S**(387) | T(2)/G(3)/D(6)/R(6)/K(15)/A(16)/S(18230)/**N**(40454) | S |
|  | 249 | **S**(393) | P(1)/R(1)/I(2)/K(7)/L(7)/A(7)/D(12)/G(16)/S(18475)/**T**(40204) | S |
|  | 312 | H(7)/**Y**(386) | V(1)/D(1)/L(2)/F(2)/I(3)/S(27)/R(80)/Y(3583)/**H**(55033) | Y |
|  | 369 | X(1)/E(1)/N(1)/**S**(390) | Y(2)/H(2)/T(2)/K(6)/D(12)/R(14)/E(38)/G(131)/S(5708)/**N**(52817) | S |
|  | 374 | F(1)/**S**(392) | Y(1)/A(1)/V(2)/I(3)/E(4)/T(15)/G(40)/F(251)/S(3393)/**L**(55022) | S |
|  | 389 | D(1)/**N**(392) | Y(1)/P(1)/I(1)/L(2)/D(2)/F(2)/H(4)/T(4)/E(12)/Q(14)/R(30)/S(41)/N(3756)/**K**(54862) | N |
|  | 404 | D(1)/K(2)/**N**(390) | K(1)/P(1)/A(1)/I(1)/H(5)/S(5)/E(6)/G(6)/Y(16)/R(40)/N(7518)/**D**(51132) | N |
|  | 439 | L(6)/**W**(387) | V(3)/T(3)/P(4)/F(8)/M(8)/S(10)/N(12)/W(1994)/**L**(56690) | W |
| M1 | 115 | A(1)/F(1)/G(2)/L(3)/M(8)/I(62)/**V**(23781) | K(1)/M(15)/V(324)/**I**(51647) | V |
|  | 121 | N(1)/S(2)/P(6)/A(237)/**T**(23612) | T(321)/**A**(51666) | T |
|  | 137 | I(1)/P(1)/G(2)/S(2)/M(3)/K(4)/D(24)/A(88)/**T**(23733) | S(1)/T(359)/**A**(51627) | T |
|  | 174 | S(2)/K(102)/**R**(23754) | R(2217)/**K**(49771) | R |
|  | 218 | N(1)/P(1)/I(4)/S(4)/A(666)/**T**(23182) | N(1)/V(13)/S(34)/T(1342)/**A**(50598) | T |
|  | 239 | D(1)/G(1)/S(1)/V(12)/T(122)/**A**(23721) | S(14)/I(16)/N(18)/A(611)/**T**(51328) | A |
| M2 | 54 | I(1)/L(7)/S(18)/H(47)/C(62)/**R**(23702) | S(3)/P(4)/X(11)/H(17)/I(59)/R(267)/F(527)/V(542)/**L**(50403) | R |
|  | 57 | C(1)/N(1)/H(81)/**Y**(23754) | R(7)/X(8)/N(77)/Q(325)/Y(352)/**H**(51064) | Y |
|  | 78 | F(1)/A(1)/P(1)/E(2)/L(5)/K(15)/H(25)/R(252)/**Q**(23535) | M(3)/N(4)/T(5)/X(9)/E(16)/R(19)/Q(270)/**K**(51507) | Q |
|  | 86 | S(1)/G(1)/L(2)/F(4)/I(7)/A(18)/**V**(23804) | D(2)/X(2)/S(6)/T(6)/V(265)/**A**(51552) | V |
|  | 93 | I(1)/K(1)/Y(6)/H(13)/T(18)/S(22)/D(36)/**N**(23740) | I(1)/L(1)/Q(1)/H(1)/X(3)/N(271)/**S**(51555) | N |
| NS1 | 41 | N(3)/R(239)/**K**(27155) | M(1)/S(4)/G(5)/K(299)/**R**(36821) | K |
|  | 82 | P(1)/G(1)/I(1)/L(1)/M(1)/S(7)/D(25)/V(78)/T(221)/**A**(22402) | T(1)/I(15)/A(1754)/**V**(35360) | A |
|  | 125 | A(1)/H(1)/B(2)/V(2)/S(7)/E(97)/G(197)/N(306)/**D**(26800) | N(1)/A(3)/K(33)/G(51)/D(236)/**E**(36806) | D |
|  | 135 | I(3)/C(4)/G(5)/R(6)/T(8)/N(52)/**S**(27336) | G(7)/S(4133)/**N**(32990) | S |
|  | 144 | I(3)**/L**(27411) | V(9)/M(16)/L(753)/**I**(36352) | L |
|  | 196 | A(1)/Q(3)/D(3)/G(3)/K(39)/**E**(27360) | N(1)/R(1)/Q(10)/E(266)/**K**(36852) | E |
| NS2 | 57 | H(1)/A(2)/P(4)/Q(4)/T(20)/Y(46)/L(71)/F(192)/**S**(21043) | J(1)/F(1)/V(1)/X(2)/Y(97)/S(138)/**L**(19078) | S |
|  | 107 | V(1)/F(2)/P(2)/**L**(21378) | V(1)/S(2)/L(123)/**F**(19192) | L |

Residues exhibiting non avian- like signatures in H3N2 CIVs was showed in blue.

Table S5 Amino acid residues separating swine from avian in H3N2

| Protein | Position | Avian residues | Swine residues |
| --- | --- | --- | --- |
| PB2 | 271 | S(1)/V(40)/X(41)/A(76)/M(83)/I(263)/**T**(22258) | S(11)/T(32)/I(284)/**A**(2174) |
|  | 456  591  645 | R(1)/I(2)/Y(6)/K(6)/X(19)/H(28)/S(195)/D(248)/**N**(22258)  X(8)/P(12)/H(34)/R(37)/L(54)/K(83)/**Q**(22535)  T(1)/F(1)/X(3)/I(5)/V(11)/L(93)/**M**(22647) | I(1)/D(20)/N(810)/**S**(1670)  S(1)/K(33)/Q(329)/**R**(2138)  M(238)/**L**(2263) |
| PB1 | 336 | A(65)/I(110)/**V**(19696) | X(2)/V(331)/**I**(1706) |
|  | 339 | L(1)/F(2)/T(3)/M(24)/V(154)/**I**(19687) | X(2)/V(2)/I(364)/**M**(1671) |
|  | 433  486  581  741 | T(1)/E(1)/H(1)/N(1)/Q(19)/R(142)/**K**(19706)  P(1)/Q(1)/G(1)/L(2)/K(107)/**R**(19759)  N(1)/K(22)/G(24)/D(321)/**E**(19503)  N(2)/G(2)/D(3)/V(19)/S(77)/T(258)/**A**(19510) | X(2)/K(400)/**R**(1637)  X(2)/R(331)/**K**(1706)  Y(2)/G(3)/L(13)/N(35)/E(344)/**D**(1642)  P(1)/V(1)/F(1)/T(9)/A(310)/**S**(1717) |
| PB1-F2 | 76 | A(110)/**V**(12518) | S(1)/V(209)/**A**(1375) |
| PA | 254  362 | D(1)/I(1)/M(1)/C(3)/K(6)/T(73)/S(90)/**N**(12000)  T(1)/N(2)/R(29)/**K**(12143) | C(1)/R(1)/N(325)/**S**(607)  K(168)/**R**(766) |
| NP | 21 | T(3)/K(11)/S(42)/D(47)/**N**(23121) | G(7)/N(391)/**D**(2191) |
|  | 119 | D(1)/M(5)/L(9)/T(10)/N(32)/V(153)/**I**(23014) | T(2)/I(476)/**V**(2111) |
|  | 189 | V(3)/L(7)/I(74)/**M**(23140) | V(16)/M(125)/L(416)/**I**(2032) |
|  | 190 | M(1)/T(2)/G(8)/I(11)/A(156)/**V**(23046) | N(1)/S(3)/I(4)/T(119)/V(410)/**A**(2052) |
|  | 289 | C(1)/L(4)/F(13)/H(113)/**Y**(23093) | Y(392)/**H**(2197) |
|  | 305 | C(1)/S(3)/H(18)/K(264)/**R**(22938) | N(98)/R(333)/**K**(2158) |
|  | 357 | E(1)/T(1)/L(3)/H(4)/R(6)/K(73)/**Q**(23136) | R(29)/Q(253)/**K**(2307) |
|  | 400 | S(1)/I(1)/W(1)/G(2)/K(592)/**R**(22627) | R(514)/**K**(2075) |
|  | 425  444  456 | M(2)/V(107)/**I**(23115)  F(1)/L(2)/T(3)/V(66)/**I**(23152)  A(10)/L(84)/M(120)/**V**(23010) | I(378)/**V**(2211)  I(555)/**V**(2034)  T(1)/M(33)/V(364)/**L**(2191) |
| M1 | 116 | T(1)/M(1)/G(1)/P(3)/S(529)/**A**(23323) | P(2)/A(630)/**S**(2506) |
|  | 209 | R(1)/P(1)/G(1)/T(67)/**A**(23788) | N(1)/A(662)/**T**(2475) |
|  | 214 | K(1)/P(1)/R(2)/H(96)/**Q**(23758) | N(1)/Q(627)/**H**(2510) |
| M2 | 77 | S(1)/P(2)/W(3)/L(6)/Q(182)/**R**(23643) | K(1)/R(641)/**Q**(2485) |
| NS1 | 125 | A(1)/H(1)/B(2)/V(2)/S(7)/E(97)/G(197)/N(306)/**D**(26800) | K(1)/A(2)/G(13)/N(27)/D(338)/**E**(2249) |
|  | 189 | E(1)/A(1)/V(1)/Y(6)/N(102)/G(110)/**D**(27188) | V(1)/C(2)/S(6)/N(8)/D(492)/**G**(2121) |
| NS2 | 32 | L(1)/M(1)/T(3)/V(82)/**I**(21296) | L(1)/I(444)/**V**(1994) |
|  | 34 | P(1)/H(2)/K(33)/L(57)/R(401)/**Q**(20889) | K(2)/Q(417)/**R**(2020) |
|  | 57 | H(1)/A(2)/P(4)/Q(4)/T(20)/Y(46)/L(71)/F(192)/**S**(21043) | C(1)/H(17)/F(42)/L(50)/S(147)/**Y**(2182) |

Table S6 Amino acid residues separating human from swine in H3N2

| Protein | Position | Swine residues | Human residues |
| --- | --- | --- | --- |
| PB2 | 120 | D(49)/**E**(2452) | A(2)/N(9)/E(506)/**D**(38441) |
|  | 199  353  475  526  567  569  627  674  682 | V(1)/D(2)/S(50)/**A**(2448)  T(1)/R(25)/**K**(2475)  M(57)/**L**(2444)  R(67)/**K**(2434)  Y(1)/S(14)/N(39)/**D**(2447)  M(2)/S(2)/A(34)/**T**(2463)  R(1)/V(3)/K(47)/**E**(2450)  G(1)/I(1)/S(5)/E(5)/T(52)/**A**(2437)  N(1)/E(1)/S(48)/**G**(2451) | Y(1)/F(1)/T(2)/A(370)/**S**(38584)  N(1)/G(1)/I(3)/K(4011)/**R**(34942)  I(9)/L(467)/**M**(38482)  K(552)/**R**(38406)  T(1)/K(9)/S(24)/D(399)/**N**(38525)  E(2)/S(93)/T(714)/**A**(38149)  T(1)/R(14)/E(376)/**K**(38567)  N(10)/I(57)/S(156)/A(442)/**T**(38293)  R(1)/I(1)/C(4)/N(96)/G(497)/**S**(38359) |
| PB1 | 576 | I(22)/**L**(2017) | V(4)/T(5)/X(8)/M(9)/L(3539)/**I**(36601) |
|  | 586  619 | X(1)/R(42)/**K**(1996)  G(13)/N(15)/E(17)/**D**(1994) | X(10)/K(3039)/**R**(37118)  A(1)/E(5)/X(6)/T(8)/K(52)/S(116)/D(2089)/**N**(37888) |
|  | 709 | I(15)/**V**(2024) | X(11)/V(1876)/**I**(38279) |
| PB1-F2 | 8  18  20  62 | S(2)/Q(2)/L(18)/**P**(1563)  T(20)/**I**(1565)  Y(1)/T(2)/R(19)/**K**(1563)  H(1)/M(1)/P(36)/**L**(1547) | H(1)/X(9)/Q(58)/P(3530)/**L**(28854)  M(1)/V(1)/A(1)/X(5)/I(5520)/**T**(26924)  E(2)/X(5)/G(6)/I(6)/S(11)/K(1938)/**R**(30484)  S(2)/X(3)/H(103)/L(997)/**P**(31347) |
| PA | 311  573 | V(1)/L(1)/I(21)/**M**(911)  V(17)/**I**(917) | X(1)/V(1)/T(2)/L(10)/M(634)/**I**(5282)  X(2)/I(954)/**V**(4974) |
| HA | 33 | K(5)/H(31)/R(70)/**Q**(3815) | Y(1)/I(2)/K(5)/D(7)/L(10)/N(15)/H(124)/Q(10419)/**R**(55959) |
| NP | 18 | G(3)/D(42)/**E**(2544) | G(3)/E(946)/**D**(39637) |
|  | 52 | C(1)/N(1)/H(10)/**Y**(2577) | L(2)/C(2)/Q(5)/N(7)/Y(2619)/**H**(37951) |
|  | 65 | K(35)/**R**(2554) | R(799)/**K**(39787) |
|  | 131 | R(2)/S(5)/**A**(2582) | T(3)/Y(6)/P(18)/R(23)/A(2776)/**S**(37760) |
|  | 214 | G(2)/K(78)/**R**(2509) | R(253)/**K**(40333) |
|  | 239 | I(5)/V(48)/**M**(2536) | A(6)/M(1370)/**V**(39210) |
|  | 280 | A(7)/I(15)/**V**(2567) | L(1)/T(5)/V(2492)/**A**(38088) |
|  | 283 | I(6)/P(62)/**L**(2521) | H(1)/Y(1)/S(2)/L(237)/**P**(40345) |
|  | 286  312  344  372  406  422  442  455  459 | V(1)/S(35)/**A**(2553)  M(4)/I(11)/**V**(2574)  L(75)/**S**(2514)  K(1)/G(2)/D(67)/**E**(2519)  T(6)/V(15)/**I**(2568)  K(64)/**R**(2525)  A(61)/**T**(2528)  N(1)/V(1)/G(1)/E(62)/**D**(2524)  K(1)/H(6)/R(61)/**Q**(2521) | A(489)/**S**(40097)  L(1)/M(13)/V(2279)/**I**(38293)  F(1)/S(241)/**L**(40344)  G(2)/N(2)/E(263)/**D**(40319)  A(1)/S(1)/I(1986)/**T**(38598)  Q(1)/M(1)/R(240)/**K**(40344)  T(243)/**A**(40343)  D(245)/**E**(40341)  W(2)/K(7)/Q(376)/**R**(40201) |
| NA | 152  217  247  249  312  389 | P(1)/Y(3)/N(8)/R(61)/**H**(3865)  T(1)/V(62)/**I**(3875)  G(1)/R(2)/N(16)/**S**(3919)  K(1)/A(3)/L(4)/Y(4)/T(39)/**S**(3887)  N(1)/F(9)/R(9)/H(90)/**Y**(3829)  S(1)/R(1)/D(1)/K(40)/**N**(3895) | N(1)/K(2)/G(3)/C(14)/D(14)/L(79)/S(179)/H(4233)/**R**(54207)  H(1)/S(3)/G(3)/T(4)/L(4)/A(5)/I(4326)/**V**(54386)  T(2)/G(3)/R(6)/D(6)/K(15)/A(16)/S(18230)/**N**(40454)  P(1)/R(1)/I(2)/A(7)/K(7)/L(7)/D(12)/G(16)/S(18475)/**T**(40204)  V(1)/D(1)/F(2)/L(2)/I(3)/S(27)/R(80)/Y(3583)/**H**(55033)  I(1)/P(1)/Y(1)/F(2)/L(2)/D(2)/T(4)/H(4)/E(12)/Q(14)/R(30)/S(41)/N(3756)/**K**(54862) |
| M1 | 115 | T(1)/I(40)/**V**(3097) | K(1)/M(15)/V(324)/**I**(51647) |
|  | 137 | K(2)/S(2)/A(56)/**T**(3078) | S(1)/T(359)/**A**(51627) |
|  | 167 | N(2)/S(3)/I(4)/A(44)/**T**(3085) | S(4)/V(17)/T(505)/**A**(51462) |
|  | 174 | M(1)/K(13)/**R**(3124) | R(2217)/**K**(49771) |
|  | 218 | S(21)/A(35)/**T**(3082) | N(1)/V(13)/S(34)/T(1342)/**A**(50598) |
|  | 230 | R(56)/**K**(3082) | K(852)/**R**(51136) |
|  | 239 | V(1)/T(48)/**A**(3089) | S(14)/I(16)/N(18)/A(611)/**T**(51328) |
| M2  NS1  NS2 | 57  78  86  89  93  28  82  135  144  196  107 | C(1)/L(1)/R(2)/H(57)/**Y**(3066)  R(2)/K(37)/**Q**(3088)  A(39)/**V**(3088)  V(1)/C(4)/D(6)/S(41)/**G**(3075)  S(40)/**N**(3087)  A(3)/N(3)/C(22)/S(40)/**G**(2562)  D(2)/V(24)/**A**(2604)  T(2)/N(8)/**S**(2620)  M(2)/I(36)/**L**(2592)  A(1)/K(73)/**E**(2556)  F(53)/**L**(2386) | R(7)/X(8)/N(77)/Q(325)/Y(352)/**H**(51064)  M(3)/N(4)/T(5)/X(9)/E(16)/R(19)/Q(270)/**K**(51507)  D(2)/X(2)/S(6)/T(6)/V(265)/**A**(51552)  I(3)/C(7)/X(10)/R(13)/N(232)/G(417)/**S**(51151)  Q(1)/H(1)/L(1)/I(1)/X(3)/N(271)/**S**(51555)  I(6)/N(108)/G(301)/**S**(36715)  T(1)/I(15)/A(1754)/**V**(35360)  G(7)/S(4133)/**N**(32990)  V(9)/M(16)/L(753)/**I**(36352)  N(1)/R(1)/Q(10)/E(266)/**K**(36852)  V(1)/S(2)/L(123)/**F**(19192) |

“X” in all above tables: vacancy, and doesn't represent any amino acid.
